# Clinical features and prognostic factors in Covid-19: A prospective cohort study

**DOI:** 10.1016/j.ebiom.2021.103378

**Published:** 2021-05-14

**Authors:** Sanne de Bruin, Lieuwe D. Bos, Marian A. van Roon, Anita M. Tuip-de Boer, Alex R. Schuurman, Marleen J.A. Koel-Simmelinck, Harm Jan Bogaard, Pieter Roel Tuinman, Michiel A. van Agtmael, Jörg Hamann, Charlotte E. Teunissen, W. Joost Wiersinga, A.H. (Koos) Zwinderman, Matthijs C. Brouwer, Diederik van de Beek, Alexander P.J. Vlaar, Amsterdam UMC COVDI-19 Biobank Investigators

**Affiliations:** a From the Department of Intensive Care, University of Amsterdam, Amsterdam UMC, Amsterdam, The Netherlands; b Department of Neurology, University of Amsterdam, Amsterdam UMC, Amsterdam Neuroscience, Amsterdam, The Netherlands; c Neurochemistry Laboratory, Department of Clinical Chemistry, Free University, Amsterdam, Amsterdam UMC, Amsterdam Neuroscience, Amsterdam, the Netherlands; d Department of Infectious Diseases, University of Amsterdam, Amsterdam UMC, Amsterdam Infection and Immunity, Amsterdam, The Netherlands; e Department of Pulmonary Medicine, Vrije Universiteit Amsterdam, Amsterdam UMC, Amsterdam Cardiovascular Sciences, Amsterdam, The Netherlands; f Department of Intensive Care, Vrije Universiteit Amsterdam, Amsterdam UMC, Amsterdam, The Netherlands; i Department of Internal Medicine, Amsterdam UMC, Vrije Universiteit Amsterdam, Amsterdam, The Netherlands; g Biobank Core Facility, Amsterdam UMC, University of Amsterdam, Amsterdam, the Netherlands; h Department of Clinical Epidemiology and Biostatistics, Amsterdam UMC, University of Amsterdam, Amsterdam Public Health, Amsterdam, the Netherlands

**Keywords:** COVID-19, Prognosis, Biomarker, Pathway analysis, Clinical features

## Abstract

**Background:**

Mortality rates are high among hospitalized patients with COVID-19, especially in those intubated on the ICU. Insight in pathways associated with unfavourable outcome may lead to new treatment strategies.

**Methods:**

We performed a prospective cohort study of patients with COVID-19 admitted to general ward or ICU who underwent serial blood sampling. To provide insight in the pathways involved in disease progression, associations were estimated between outcome risk and serial measurements of 64 biomarkers in potential important pathways of COVID-19 infection (inflammation, tissue damage, complement system, coagulation and fibrinolysis) using joint models combining Cox regression and linear mixed-effects models. For patients admitted to the general ward, the primary outcome was admission to the ICU or mortality (unfavourable outcome). For patients admitted to the ICU, the primary outcome was 12-week mortality.

**Findings:**

A total of 219 patients were included: 136 (62%) on the ward and 119 patients (54%) on the ICU; 36 patients (26%) were included in both cohorts because they were transferred from general ward to ICU. On the general ward, 54 of 136 patients (40%) had an unfavourable outcome and 31 (23%) patients died. On the ICU, 54 out of 119 patients (45%) died. Unfavourable outcome on the general ward was associated with changes in concentrations of IL-6, IL-8, IL-10, soluble Receptor for Advanced Glycation End Products (sRAGE), vascular cell adhesion molecule 1 (VCAM-1) and Pentraxin-3. Death on the ICU was associated with changes in IL-6, IL-8, IL-10, sRAGE, VCAM-1, Pentraxin-3, urokinase-type plasminogen activator receptor, IL-1-receptor antagonist, CD14, procalcitonin, tumor necrosis factor alfa, tissue factor, complement component 5a, Growth arrest–specific 6, angiopoietin 2, and lactoferrin. Pathway analysis showed that unfavourable outcome on the ward was mainly driven by chemotaxis and interleukin production, whereas death on ICU was associated with a variety of pathways including chemotaxis, cell-cell adhesion, innate host response mechanisms, including the complement system, viral life cycle regulation, angiogenesis, wound healing and response to corticosteroids.

**Interpretation:**

Clinical deterioration in patients with severe COVID-19 involves multiple pathways, including chemotaxis and interleukin production, but also endothelial dysfunction, the complement system, and immunothrombosis. Prognostic markers showed considerable overlap between general ward and ICU patients, but we identified distinct differences between groups that should be considered in the development and timing of interventional therapies in COVID-19.

**Funding:**

Amsterdam UMC, Amsterdam UMC Corona Fund, and Dr. C.J. Vaillant Fonds.

**Research in context**

**Evidence before this study**

Patients with severe COVID-19 are characterized by hyper-inflammation and coagulopathy. Studies have evaluated plasma biomarkers for inflammation and coagulopathy as prognostic factors for outcome in COVID-19. Many of these studies were retrospective, included a limited number of markers, or were limited to markers measured on admission only. We searched PubMed, EMBASE and Cochrane Reviews for research articles from January 1 2020 to February 1, 2021. More than 200 observational cohorts were published describing prognostic factors for severity of COVID-19. Only few of these studies reported dynamic changes of a very limited set of specific biomarkers; changes in interleukin (IL)-6 and D-dimer were related to clinical outcome.

**Added value of this study**

In this large prospective cohort of mild and severe hospitalized COVID-19 patients, serial prognostic biological factors were determined including blood molecular markers of endothelium activation, inflammation, neutrophil activation and neutrophil extracellular traps formation, complement activation, coagulopathy and epithelial barrier disruption. This study shows that endothelial dysfunction is a key characteristic in COVID-19 patients admitted to the ward. Patients with mild COVID-19 progressing to severe COVID-19 needing ICU admission show involvement of inflammation, coagulation, complement and epithelial barrier disruption on top of endothelial dysfunction. This study shows the key pathways involved in the progression of severe COVID-19 and may guide the way to interventional trials in severe COVID-19.

**Implications of all the available evidence**

Temporal differences between general ward and ICU patients should be taken account when designing interventional studies for severe COVID-19. On the general ward early interventions should focus on enhancing endothelial integrity and limiting chemotaxis. For intubated patients on the ICU, therapeutic interventions in multiple pathways may be needed. However, for single interventions those treatment with a day function within the crossroads of inflammation and coagulation might be most promising.

Alt-text: Unlabelled box

## 1 Introduction

Severe acute respiratory syndrome coronavirus 2 (SARS-CoV-2) causes COVID-19, a respiratory disease with high clinical variability [1]. Clinical risk factors for developing life-threatening disease are male gender, older age and comorbidities such as diabetes mellitus and hypertension [1]. Host genetic variants of interferon immunity and presence of auto-antibodies against type I interferon also have been identified as risk factors [2], [3], [4]. Patients hospitalized with COVID-19 predominantly present with hypoxemia caused by virus-induced lung inflammation characterized by lymphocyte infiltration and activation of the coagulation system [5]. As a result, 20-40% of the hospitalized COVID-19 patients require ICU admission, [6] and 35-50% of these patients have a fatal outcome [7]. The wide range between rate of ICU admission and death can be explained by various reasons, most importantly differences in the threshold for hospital admission.

Biomarkers, mainly blood chemistry markers, [8] have been associated with outcome in COVID-19. However, many of these studies were retrospective, evaluated a limited number of markers, or only evaluated markers measured on admission [8]. So far, dynamic changes of a few biomarkers, including interleukin (IL)-6 and D-dimer, have been associated with outcome in large datasets [9,10]. While it has been postulated that secondary damage in COVID-19 is caused by a “cytokine storm”, systemic levels of pro-inflammatory cytokines are generally lower than reported in patients with acute respiratory distress syndrome (ARDS) from aetiologies other than corona virus [11,12]. Autopsy reports in COVID-19 suggested that respiratory failure is also driven by other pathways than the “cytokine-storm-pathway”, for example, endothelial activation, neutrophil activation and neutrophil extracellular trap (NET) formation, immunothrombosis, and epithelial barrier disruption [13,14].

So far, adjunctive dexamethasone therapy has been shown to prevent mortality in severe COVID-19, while monoclonal antibodies against IL-6 showed ambiguous results in clinical trials [[15], [16], [17], [18], [19],^20^]. One study preprint reported that monoclonal antibodies against IL-6 improved survival and other clinical outcomes in hospitalised COVID-19 patients with hypoxia and systemic inflammation [21]. Among those not receiving invasive mechanical ventilation at baseline, patients allocated tocilizumab were less likely to reach the composite endpoint of invasive mechanical ventilation or death [21]. Optimal patient selection and timing of treatment must be considered crucial to optimize adjunctive treatments for COVID-19. We performed a prospective cohort study with serial sampling to evaluated the importance of multiple biomarkers in potential pathways over time in severe COVID-19.

## 2 Methods

### 2.1 Study design

The Amsterdam UMC COVID-19 biobank is a prospective cohort study containing clinical data and archive material from adult patients admitted with COVID-19 in two academic hospitals of Amsterdam UMC. Based on previous studies, 64 blood molecular markers of endothelial activation, inflammation, neutrophil activation and NET formation, activation of the complement, coagulopathy and epithelial barrier disruption, were measured in serial blood archive samples from patients admitted from 23^rd^ of March until 26^th^ of May 2020.

### 2.2 Ethics

Patients or their legal representatives received written information about the study and were asked to give written informed consent for participation. If direct informed consent of patients was not feasible, patients could be included with a deferred consent procedure. In case of deferred consent, patients or their legal representatives were informed about the biobank as soon as possible. To ensure all patients wilfully participated in the biobank and provide a possibility to opt out from the biobank comprehensive information and an opt-out form was send to the patients three months after discharge. This study was approved by the biobank ethics committees of both Amsterdam UMC hospitals (2020_065).

### 2.3 Patient selection

Patients admitted to one of the two academic hospitals within Amsterdam UMC were prospectively included if they were admitted on the COVID-19 ward (general ward cohort), COVID-19 ICU (ICU cohort) or on the regular ICU (control ICU cohort). A definite COVID-19 case was defined as a positive PCR from nasopharyngeal swab with clinical signs consistent with COVID-19. For the current analysis, patients were selected when a blood sample was available within 2 days of ward- and/or ICU-admission. So, patients were excluded if there was no blood sample available. Patients were treated according to the local protocol that included thromboprophylaxis, but did not routinely include the use of remdesivir, hydrochloroquine, azithromycin, convalescent plasma, corticosteroids or any other immunomodulatory therapy. High flow nasal cannulae were not used during the inclusion period due to initial concerns for the safety of healthcare workers. Invasively ventilated patients received low tidal volume ventilation with PEEP set to the lower PEEP-FiO2 table and mandatory prone positioning at a PaO2/FiO2 below 150 mmHg.

### 2.4 Data collection

Clinical data were collected using the case record form (CRF) of the World Health Organisation (WHO) [22]. The day of admission to the general ward or to the ICU was identified as timepoint 0. Subsequent timepoints were defined up to 28 days after admission (so day 0-2, followed by day 3-4, day 5-6, day 9-10, day 13-15, day 20-22, day 27-29). Biomarkers were measured using a luminex platform or ELISA (online appendix eTable 1). Plate-to-plate variation was accounted for using negative and positive controls. Values below the detection limit were imputed with the lower limit of quantification given by the calibration curve for the univariate comparisons, but were not taken into account in joint model analysis (see statistical analysis paragraph). More than 50% of the ferritin, endothelin-1, interleukin (IL)-1 beta and IL-12-p70 measurements were judged to be unreliable because of stringent quality criteria (more than 25 beads counted and no extrapolation outside of the reference standard concentrations) and were therefore excluded for analysis.

**Table 1:** Baseline demographics.

Characteristics	General ward cohort (N=136)			ICU cohort (N=119)		
	Favourable outcome (N=82)	Non-favourable outcome (N=54)	p-value	Survivors (N=74)	Non-survivors (N=45)	p-value
Male	50 (61%)	36 (67%)	0.62	53 (72%)	31 (69%)	0.91
Age, years	63.7 (12.8)	65.5 (11.1)	0.40	60.1 (10.5)	64.6 (10.1)	0.02
Body mass index (BMI)	28.7 [25.9, 31.5]	26.9 [24.2, 31.5]	0.17	27.0 [24.8, 31.0]	27.7 [25.9, 30.0]	0.87
Do not resuscitate order at hospital admission n (%)	19 (23%)	9 (17%)	0.24	7 (10%)	8 (18%)	0.28
Do not intubate order at hospital admission (%)	15 (18%)	8 (15%)	0.40	1 (1%)	0 (0%)	0.36
Oxygen support during hospitalisation (%)	73 (89%)	54 (100$)	0.03	74 (100%)	45 (100%)	NA
						
**Medical history**						
Hypertension	40 (49%)	23 (43%)	0.66	30 (41%)	21 (47%)	0.69
Diabetes without complications	16 (20%)	14 (26%)	0.50	17 (23%)	13 (29%)	0.62
Diabetes with complications	8 (10%)	3 (6%)	0.58	5 (7%)	1 (2%)	0.51
Chronic Pulmonary Disease	11 (13%)	4 (7%)	0.42	5 (7%)	7 (16%)	0.22
Asthma	7 (9%)	3 (6%)	0.77	15 (20%)	2 (5%)	0.04
Chronic Kidney Disease	9 (11%)	5 (9%)	1.00	3 (4%)	2 (4%)	1.00
Liver disease	3 (4%)	1 (2%)	0.93	2 (3%)	1 (2%)	1.00
Chronic Neurological Disease	13 (16%)	12 (23%)	0.45	6 (8%)	6 (14%)	0.52
Active Solid Malignancy	5 (6%)	3 (6%)	1.00	1 (1%)	4 (9%)	0.12
Active Haematological Disease	1 (1%)	3 (6%)	0.34	2 (3%)	4 (9%)	0.27
Usage of immunosuppressive agents prior to admission	6 (7%)	6 (11%)	0.63	7 (9%)	1 (2%)	0.24
Rheumatological disorders	10 (12%)	4 (7%)	0.54	4 (5%)	2 (4%)	1.00
**Laboratory values**						
White blood cell count (x10^9/L)	8.07 (3.72)	6.93 (2.64)	0.044	8.99 (3.97)	8.77 (3.81)	0.77
Lymfocytes (x10^9/L)	0.88 [0.65, 1.27]	1.05 [0.75, 1.35]	0.33	0.96 [0.69, 1.26]	0.83 [0.47, 1.83]	0.79
Platelets (x10^9/L)	237 (95)	253 (104)	0.40	274 (107)	246 (94)	0.16
C reactive protein (mmol/L)	125 (98)	94 (60)	0.035	155.08 (99.56)	186.26 (125.77)	0.24
D-dimer (mg/L)	1.24 [0.93, 1.87]	1.14 [0.90, 1.81]	0.71	1.08 [0.78, 2.17]	4.72 [1.26, 13.13]	0.03
Creatinin (mmol/L)	86 [73, 106]	83 [66, 113]	0.60	77 [63, 113]	91 [74, 124]	0.12
LDH (mmol/L)	442 (191)	382 (208)	0.19	437 (190)	459 (173)	0.68
SGOT (mmol/L)	47 [35, 86]	47 [35, 80]	0.99	62 [42, 89]	55 [39, 63]	0.14
**Complications during hospital admission**						
Pulmonary embolism	5 (6%)	16 (30%)	0.001	24 (32%)	12 (27%)	0.65
Bacterial pneumonia	5 (6%)	12 (22%)	0.01	9 (12%)	16 (36%)	0.005
Renal replacement therapy	0 (0%)	9 (17%)	0.001	13 (17%)	11 (24%)	0.50
Mortality	0 (0%)	31 (57%)	<0.001	0 (0%)	45 (100%)	NA

### 2.5 Endpoints

The analysis was stratified for patients admitted to the general ward and to the ICU. For patients admitted to the ward, the primary outcome was admission to the ICU or mortality (referred to as unfavourable outcome). For patients admitted to the ICU, the primary outcome was 12-week mortality. Kaplan-Meier curves for the risk of unfavourable outcomes are reported in the supplement material. Secondary outcomes were severe complications of COVID-19 infection: pulmonary embolism, vascular thrombotic events and acute kidney failure defined by the need for renal replacement treatment (only for ICU patients). Patients who clinically deteriorated routinely underwent pulmonary CT angiography to rule out pulmonary embolism. COVID-19 patients were screened for vascular thrombotic events with duplex ultrasound once weekly.

### 2.6 Statistics

All analyses were performed in R-statistics through the R-studio interface. Continuous data was summarized with mean and standard deviation or median with inter-quartile range (IQR) dependent on the distribution of values. Categorical data were summarized with numbers and percentages. Differences between groups at baseline were tested with a T-test, Mann-Whitney U or Fisher exact test where appropriate.

The associations between outcome risk and biomarker-values were estimated using joint models that combine Cox regression and linear mixed-effects (LME) models [23]. With the LME part of the joint models we estimated the linear change pattern of the biomarkers over follow-up time. We used the assumption that both the intercepts and the slopes of follow-up time varied between patients if indicated based on the Akaike Information Criterion (AIC). Absolute biomarker concentrations and fold changes in those concentrations over time were illustrated with boxplots, stratified per cohort and outcome. The log Hazard Ratios (HRs) of the time-dependent biomarker-values for outcome were estimated with the Cox regression part of the joint models and the Weibull-specification was used to describe the cumulative baseline hazard curve [23]. These analyses were done in the ward and ICU cohort separately for the 60 biomarkers with data of sufficient quality. For three biomarkers in the ward cohort (complement component 3a, C-reactive protein and Plasminogen activator inhibitor-1) the joint models did not converge and a Cox regression model with time-dependent co-variates was used instead. To evaluate the baseline risk for outcomes, HRs of baseline biomarker values and of clinical risk factors at day of admission were estimated using Cox regression. All HRs were adjusted for age and gender of the patients. In order to evaluate the association between biomarker concentration at baseline and the occurrence of severe complications (pulmonary embolism, thrombotic vascular events and kidney failure) multinomial regression was performed and the odds ratios (ORs) were reported for occurrence of a complication and mortality. Missing baseline data was imputed using the linear mixed effect model for this analysis.

All biomarkers were [2] log-transformed in order to normalize their distributions. As a consequence, the reported Hazard Ratios must be interpreted as effects on outcome-hazard upon a doubling of the biomarker. Statistical significance was defined after Bonferroni-correction for the number of analysed biomarkers (p-value less than 0•05 divided by 60).

For pathway analysis, the biomarkers were matched to their corresponding genes via the NCBI gene library. Biomarkers that were significantly associated with outcome in the joint model analyses were used for gene ontology enrichment analysis (*enrichGO* algorithm from the *clusterProfiler* package), [24] using list of all measured biomarkers mapped to the human genome as reference. Significant pathways were defined by a P-value below 0•01 after Bonferroni correction with a Q-value below 0•05.

### 2.7 Role of funders

This study was funded by the Amsterdam UMC, Amsterdam UMC Corona Fund, and Dr. C.J. Vaillant Fonds (to DB). Funders had no role in the in study design, data collection, data analyses, interpretation, or writing of this report.

## 3 Results

Between March 23^rd^ and May 26^th^ 2020, 416 patients with COVID-19 were included in the Amsterdam UMC COVID-19 Biobank (online appendix eFig. 1). From 200 of the 417 patients (48%), no blood sample was present on the first day, resulting in 219 (52%) patients eligible for the current analysis. The 219 selected patients were were older and more frequently male and had a more severe clinical course defined by increased thromboembolic events and mortality (online appendix eTable 2). Of the 219 patients included, 136 (62%) were initially managed on the ward and 119 patients (54%) were admitted to the ICU. 36 patients (26%) were transferred from the ward to the ICU and were included in both cohorts.

**Fig. 1:**
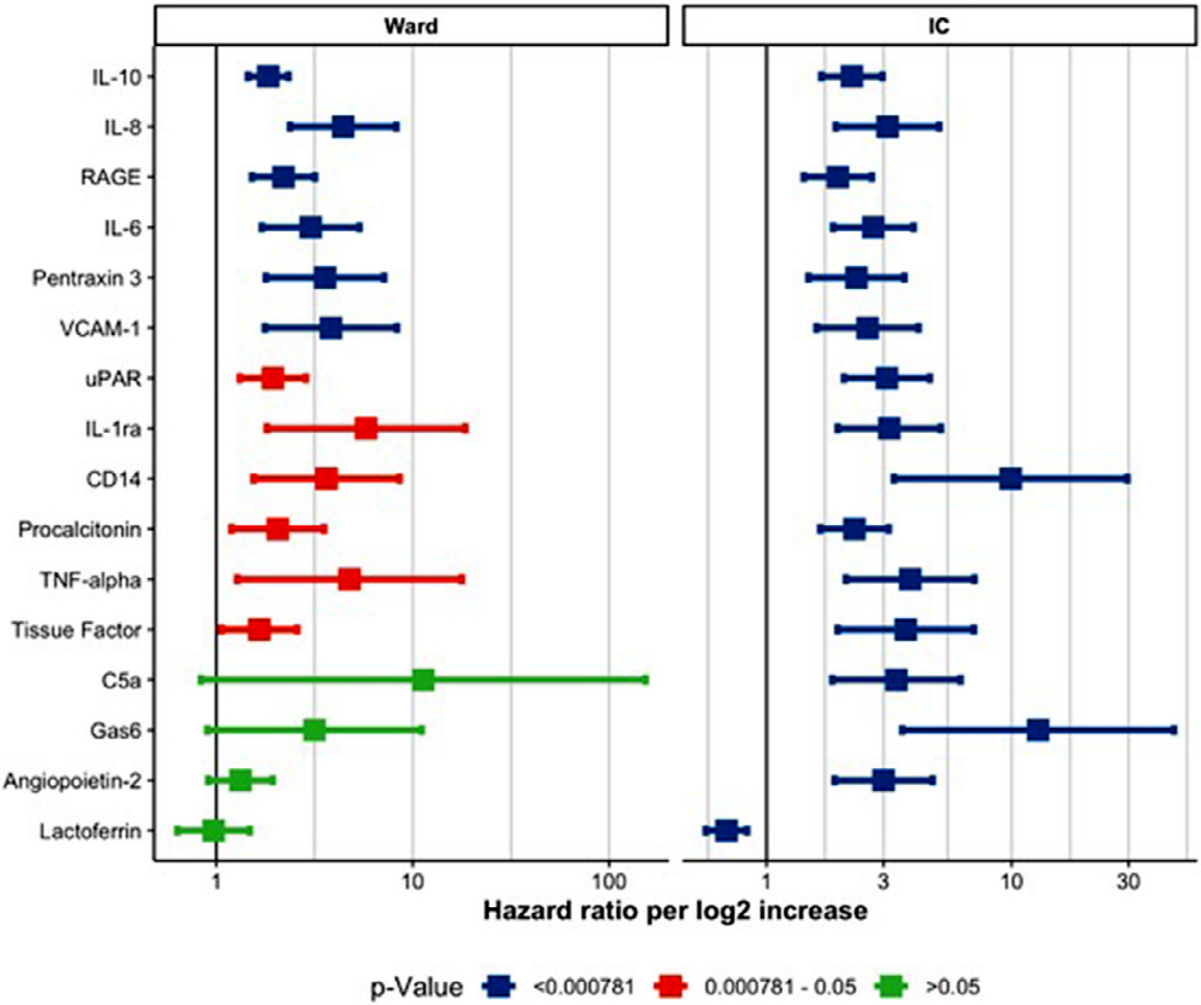
Forrest plot for hazard ratio per log2 increase in biomarker value stratified per cohort. legend. X-axis shows the hazard ratio per log2 increase in biomarker concentration. The square indicates the estimated effect while the whiskers indicate the 95% confidence interval. The colours indicate the p-value corresponding with the confidence interval. Blue lines indicate biomarkers with a P-value below 0•05 after Bonforroni correction. Red lines indicate biomarkers with a P-value that was below 0•05 before adjustment, but did not reach statistical significance after Bonforroni correction. Green lines indicate non-significant biomarkers. The current Fig. only shows biomarker with an adjusted P-value below 0•05 in either cohort. Supplemental Figs. 4 and 5 show all biomarkers. (For interpretation of the references to colour in this figure legend, the reader is referred to the web version of this article.)

Median age of general ward COVID-19 patients was 64 years (SD 12•1; Table 1) of whom 86 out of 136 were men (63%). Median age of ICU COVID-19 patients was 62 years (SD 10•6) of whom 84 out of 119 were men (71%). 25 ward patients (20%) were admitted with a do not resuscitate order, and 23 patients (18%) with a do not intubate order. Most patients had coexisting risk-associated conditions, most commonly hypertension (102 [47%]), diabetes (53 [24%]), and chronic pulmonary (26 [12%]) or neurological disease (31 [14%]).

The overall case fatality was 59 of 217 patients (27%; Table 1). On the ward, 54 of 136 patients (40%) had an unfavourable outcome and 31 (23%) patients died. On the ICU, 54 out of 119 patients (45%) died. Kaplan–Meijer curves are shown in the online appendix eFigure2. Complications occurred in a high proportion of patients: pulmonary embolism in 41 of 217 (19%; 21 of 136 ward patients [15%], 36 of 119 ICU patients [30%]) and bacterial pneumonia in 30 of 217 (14%; 17 of 136 ward patients [13%], 25 of 119 ICU patients [21%]). Pulmonary aspergillosis was diagnosed in 13 of 119 ICU patients [11%], and 24 of 119 ICU patients [20%]) required renal replacement therapy.

For patients on the general ward, we used 26 age-matched controls from the outpatient clinic, with a mean age of 64 years (SD 15•5; online appendix eTable 3) of whom 18 (69%) were male. For ICU COVID-19 patients, controls were patients admitted on the ICU who did not have COVID-19 disease or other viral respiratory illness, with a median age of 60 years (SD 17•6; online appendix eTable 4) of whom 17 were men (68%). Most common admission diagnoses were respiratory failure (6 of 25 [24%]) and post-surgical care (7 of 25 [28%]).

**Fig. 2:**
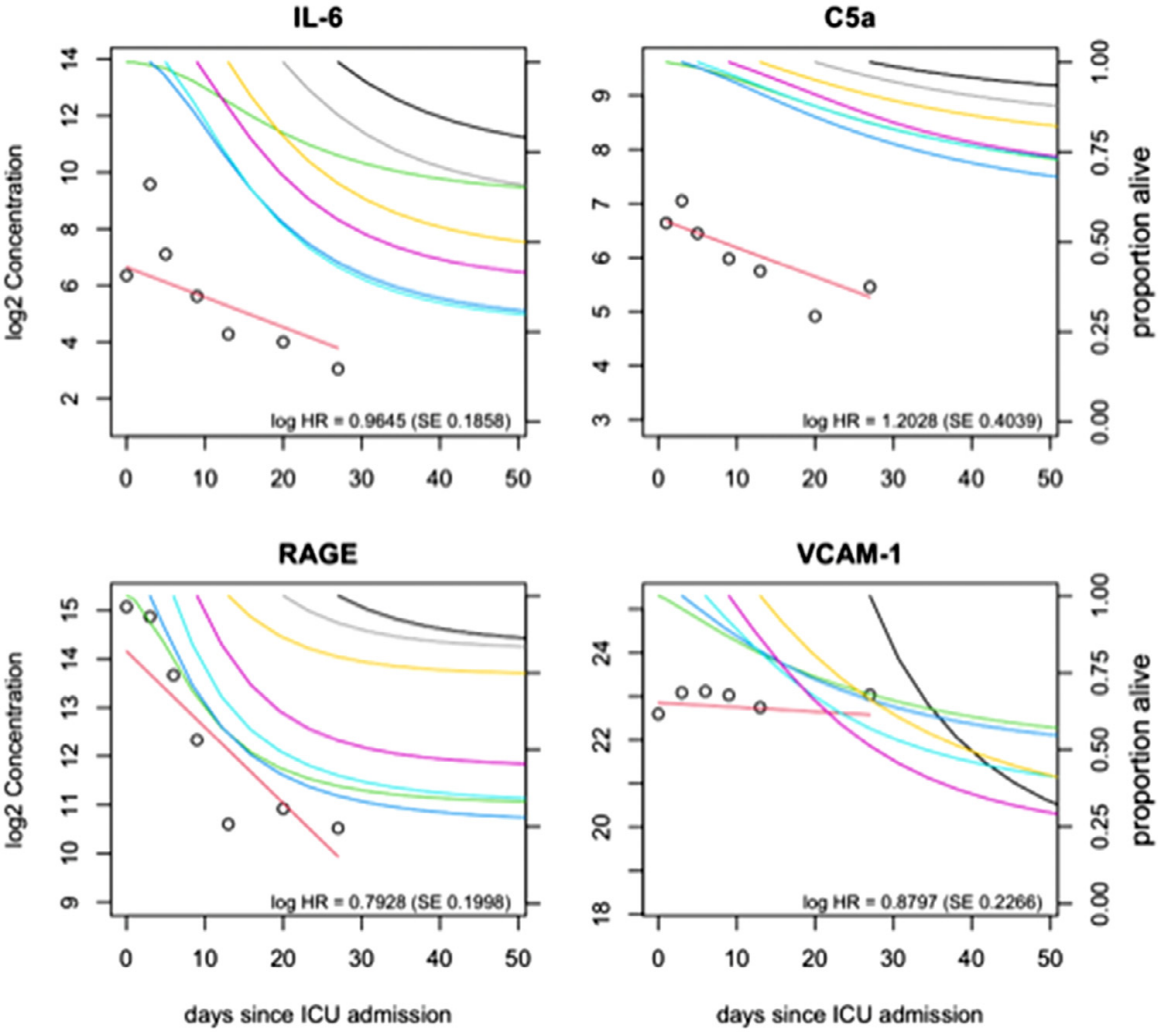
Dynamic changes of important biomarkers in four patients that examplify the change in prognosis based on longitudinal biomarker information modeled by joint model analysis. legend: Four plots illustrating the change of four markers since day of ICU admission in four different patients. Panel A shows the seven available IL-6 blood levels measurements for the first patient (male, 63 yr). At day of admission IL-6 level was close to the average baseline IL-6 levels in our sample and therefore the predicted survival-curve at ICU admission for this patient (green line) was close to the observed Kaplan-Meier curve for the entire sample of patients. At days 3 and 5 of ICU admission IL-6 levels were increased compared to baseline and the predicted survival-curves at these days (dark and light blue curves) indicated substantial worsening of the prognosis. At days 9, 13, 20 and 27 IL-6 levels were decreased in this patient and the predicted survival-curves at these days (purple, yellow, grey and black curves) indicated clear improvement of the prognosis for this patient who was discharged alive from the ICU at day 38. Panels B and C illustrate similar change patterns of the C5a and RAGE markers in two other patients (male, 59 yr and male 74 yr), who were discharged alive from the ICU alive after 48 and 44 days, respectively. Panel D illustrates the change pattern of six measurements of the VCAM-1 blood levels in a 74 yr old male patient. In contrast to what was observed in other patients, the VCAM-1 measurements of this patient remained relatively stable at an increased level during his ICU stay, and a progressive worsening of the predicted survival-curves was seen (green, blue, purple, yellow, black curves). The patient died at day 47 after ICU admission. (For interpretation of the references to colour in this figure legend, the reader is referred to the web version of this article.)

**Fig. 3:**
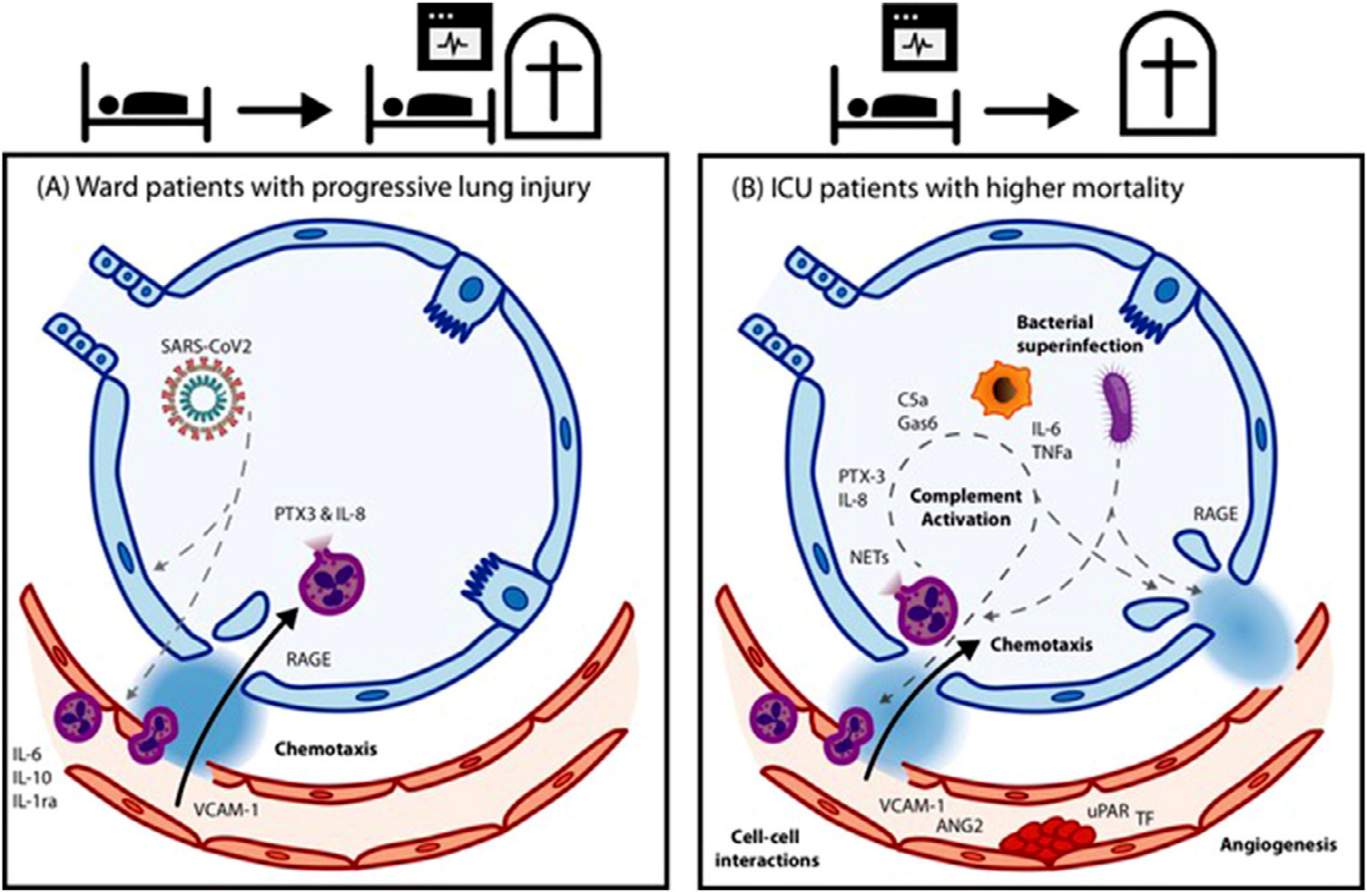
Schematic overview of the pathophysiological processes involved in clinical deterioration in ward (left) and ICU patients with Covid-19 (right).

Several plasma biomarkers were associated with outcome (Figs. 1 and 2; online appendix eTable 3 and eFig. 4-9). On the general ward, changes in concentrations over time (comparison between the first blood with subsequent blood samples) of IL-6, IL-8, IL-10, soluble Receptor for Advanced Glycation End Products (sRAGE), vascular cell adhesion molecule 1 (VCAM-1) and Pentraxin-3 (PTX3) were associated with unfavourable outcome. On the ICU, changes in concentrations over time (first blood in ICU and then subsequent blood samples) of IL-6, IL-8, IL-10, sRAGE, VCAM-1, Pentraxin-3, urokinase-type plasminogen activator receptor (UPAR), IL-1-receptor antagonist (IL-1RA), CD14, procalcitonine, tumor necrosis factor alfa (TNFa), tissue factor (TF), complement component 5a (C5a), Growth arrest–specific 6 (GAS-6), angiopoietin 2 (ANG2), and lactoferrin were associated with death. Fig. 3 shows the individual biomarker trajectories of four individual patients and highlights the change in prognosis based on the dynamic biomarker profiles. The association between each biomarker and outcome at baseline alone is reported in the online appendix eFig. 10. Temporal changes in the plasma concentration of CRP and PAI-1 showed a stronger association with adverse outcome in patients admitted to the normal ward when corrected for BMI in a sensitivity analysis (online appendix eFig. 11). The same analysis for patients admitted to the ICU showed a stronger association for TFF3 but a weaker association for MPO (online appendix eFig. 12).

Baseline biomarker concentrations were associated with pulmonary embolism, thrombotic vascular events and kidney failure, and with unfavourable outcome on the general ward and death on the ICU (online appendix eFig. 13, 14, and 15). The biomarkers showed a similar prognostic value for pulmonary embolism and thrombotic vascular events as for death. However, the association between kidney failure and baseline concentrations for Cystacin C (OR 3•89, 95%-CI: 1•80–8•39), Neutrophil gelatinase-associated lipocalin (NGAL; OR 2•81, 95%-CI: 1•53–5•20), Trefoil factor 3 (TFF3; OR 12•2, 95%-CI: 3•93–38•0), TNF- receptor inhibitor (TNFRI; OR 6•51, 95%-CI 2•81–15•1) and Triggering receptors expressed on myeloid cells-1 (TREM-1; OR 5•52, 95%-CI: 2•46–12•4) was stronger than the associations between these markers and death.

Pathway analyses revealed that on the general ward biomarkers associated with outcome were mainly mapped to chemotaxis and interleukin production (Fig. 4, online appendix eTable 4). For the ICU patients, there was a much wider variety of pathways involved which included chemotaxis, cell-cell adhesion, innate host response mechanisms, including the complement system, viral life cycle regulation, angiogenesis, wound healing and response to corticosteroids (online appendix eTable 5)**.**

## 4 Discussion

In our prospective study with serial sampling, we evaluated the combination of multiple biomarkers in potential pathways involved in COVID-19. Our results show that deterioration in patients with hospitalized COVID-19 involves multiple pathways, including chemotaxis and interleukin production, but also endothelial dysfunction, the complement system, and immunothrombosis. Identified prognostic markers showed considerable overlap between patients on the ward and ICU, but also distinct differences. On the general ward the strongest predictors were indicative of endothelial activation and chemotaxis. On the ICU, enhanced involvement of inflammation, activation of the complement system and coagulation, and further breaching of the epithelial barrier were found as markers of poor prognosis. Evaluated markers may not indicate a direct causal relationship, but our findings have implications for development and timing of interventional therapies in severe COVID-19.

Our findings implicate that therapeutic interventions in patients with COVID-19 on the general ward, so early after hospital admission, should aim to improve endothelial integrity and limit chemotaxis towards the alveolar compartment. Concentrations of IL-6 were associated with unfavourable outcome for ward and ICU patients. Tocilizumab is a humanized IL-6 receptor-inhibiting monoclonal antibody for the rheumatoid arthritis giant cell arteritis, systemic juvenile idiopathic arthritis and for people with chimeric antigen receptor T cell-induced severe or life-threatening cytokine release syndrome. Ten randomized controlled studies on anti-IL6 treatment in hospitalized patients with COVID-19 have been reported [[17], [18], [19], [20], [21],[25], [26], [27], [28]]. These studies showed conflicting data regarding the use of anti-IL6 for COVID-19 patients, which may be explained by the additional use of dexamethasone, the timing of start of anti-IL6 in the disease course and patient selection. Our results may help to optimize the timing of anti-IL6 as well as the selection of patients which may benefit most.

Imatinib is one of the drugs that reinforce the endothelial barrier and mitigate alveolar inflammatory responses through NFkB mediated chemotaxis in several models of acute lung injury and may therefore prevent clinical progression of COVID-19 [29]. On an individual level, stable VCAM-1 concentration over time were strongly associated with progressive worsening of the predicted survival-curves. At least two randomized controlled studies are ongoing that test this intervention in hospitalised patients with COVID-19 (EUDRACT2020-005447-23) [30]. Other drugs that could limit chemotaxis and endothelial dysfunction are blockers of RAGE and transient receptor potential vanilloid 4 (TRPV4) channel inhibitors [31,32]. These drugs are currently not registered as being tested in randomized controlled studies for COVID-19.

Our study confirms the key role of hyperinflammation and immunothrombosis in severe COVID-19 on the ICU. Some patients are at risk to develop severe COVID-19, which can partly be explained by presence of the risk factors male gender, older age and comorbidities. Recent studies have provided insight why patients with severe COVID-19 develop hyperinflammation, among others due to improper IgG glycosylation, auto-antibodies against IFN and Annexin A2, and genetic polymorphisms [2,3,33,34]. The occurrence of hyperinflammation, illustrated by enhancing levels of IL-6, IL-8, and sRAGE, explains the biphasic pattern in COVID-19, with an early viral replication phase, followed by a hyper-inflammatory phase involving cytokine release [35]. Identified markers in our study go beyond cytokine release, with PTX3, GAS6, and C5a. PTX3 is an essential component of humoral innate immunity, involved in resistance to selected pathogens and in the regulation of inflammation [36]. RNA-sequencing analysis of peripheral blood mononuclear cells, single-cell bioinformatics analysis and immunohistochemistry of lung autopsy samples revealed that myelomonocytic cells and endothelial cells express high levels of PTX3 in patients with COVID-19 [36].

The cumulative response of the immune system to SARS-CoV-2, both through inflammation and immune cell expression of prothrombotic proteins, is likely to be a major contributor to hypercoagulability in COVID-19. Recognition of pathogen-associated molecular patterns through the toll like receptors and CD14 receptor of monocytes promotes the transcription and expression of TF [37]. Neutrophils of COVID-19 patients yielded high TF expression and released NETs carrying active TF [38]. Treatment of control neutrophils with COVID-19 platelet-rich plasma generated TF-bearing NETs that induced thrombotic activity of endothelial cells [38]. Thrombin or NETosis inhibition or C5a blockade attenuated platelet-mediated NET-driven thrombogenicity. The association of COVID-19 inflammation with activation of the C5a–C5aR1 axis has been reported [39]. The potent anaphylatoxin C5a attracts neutrophils and monocytes to the infection site, and strongly activates these cells, causing tissue damage by oxidative radical formation and enzyme release but also inducing release of tissue factor from endothelial cells and neutrophils thereby activating the coagulation system [40]. A phase 2 randomized controlled trial show that C5a inhibition was safe and associated with decreased risk for pulmonary embolisms in severe COVID-19 patients [40]. The GAS6-PROS1/TAM system has been suggested to play an important role in SARS-CoV-2 infection and progression complications [41]. Dysregulation of the urokinase receptor system (UPAR) have been associated with the development of immunothrombosis associated with respiratory failure in COVID-19 patients [45].

Our study has limitations. First, we studied hospitalized patients with COVID-19. Patients with mild complaints were not taken into consideration in this analysis. This limits our conclusions to patients with severe COVID-19 disease admitted to the hospital. Second, we evaluated selected patients from the Amsterdam UMC Covid-19 Biobank. Selection was made on the basis of plasma sample availability on the first admission day. Although selected patients were comparable to non-selected patients, they tended to have more comorbidities and a more severe clinical course. This could well be explained by inclusion of relatively higher proportion of severe patients, who had a relatively high frequency of blood withdrawals. Third, we included patients from two academic hospitals in the Netherlands. Nevertheless, we believe the cohort is representative of and for the wider population. Of course, in a “normal” academic setting a potential bias maybe introduced this way. For example, transfers often have missing data and they will be excluded this way. In a normal setting transfers are often representative for complex academic cases. In the setting of COVID-19 care no transfers were made for academic expertise. The only reason for inter-hospital transfers in the Netherlands (and the same holds true for other European countries) was capacity issues. So, we believe the cohort is representative of and for the wider population. Fourth, we did not evaluate ethnicity in the current study, so we could not include this in the prognostic model. Fifth, identified markers may not indicate a causal relationship. Higher levels of IL-10 were associated with unfavourable outcome. IL-10 is considered anti-inflammatory, but has been noted to be higher in sepsis patients, proposed the increase might be an attempt to moderate the immune response. Sixth, our patients were included in the first wave of COVID-19 in the Netherlands. Ever since treatment has involved, including the use of anticoagulants and adjunctive dexamethasone therapy. The impact on outcome of the evolving treatment of COVID19 has been limited, but nevertheless, the introduction of steroids might have impacted prognostic factors. Detailed data on treatment is being collected but currently not available for the analysis. Finally, some potential important biomarkers were not included. For example, we did not evaluate troponin that has been associated with complications in COVID-19 patients [42,43,44]. We aimed to provide insight in pathways associated with unfavourable outcome, and did evaluate the additive prognostic effect of biomarkers on known clinical prognostic factors in COVID-19.

Our results may explain why single pathway intervention studies in severe COVID-19 so far remain negative or have limited impact on outcome, and only a general broad intervention on inflammation such as steroids showed benefit [[15], [16], [17], [18], [19], [20],[24], [25], [26], [27]]. We did not evaluate which markers were the best independent biomarkers predicting unfavorable outcome. However, we identified multiple pathways to be important in the pathophysiology of severe COVID-19. On the general ward early interventions should focus on enhancing endothelial integrity and limiting chemotaxis. For intubated patients on the ICU, therapeutic interventions in multiple pathways may be needed. However, for single interventions those treatment with a day function within the crossroads of inflammation and coagulation might be most promising.

## Contributors

Sanne de Bruin: study design, data collection, data analysis, data interpretation, writing

Lieuwe D. Bos:s tudy design, data collection, data analysis, data interpretation, writing

Marian A. van Roon: data collection, data analysis, data interpretation

Anita M. Tuip-de Boer: data collection, data analysis,

Alex R. Schuurman: data collection, data analysis,

Marleen J.A. Koel-Simmelinck: data collection, data analysis

Harm Jan Boogaard: data collection, writing

Pieter Roel Tuinman: data collection, writing

Michiel A. van Agtmael: study design, data collection, writing

Jörg Hamann: study design, data collection, writing

Charlotte E. Teunissen: study design, data collection, data analysis, writing

Joost Wiersinga: study design, data collection, writing

A.H. (Koos) Zwinderman: study design, data analysis, writing

Matthijs C. Brouwer: study design, data collection, data analysis, writing

Diederik van de Beek: study design, data collection, data analysis, writing, funding, principle investigator

Alexander P.J. Vlaar: study design, data collection, data analysis, writing, funding

Authors for the Amsterdam UMC Covid-19 Biobank project: data collection

All the authors had full access to the data, vouch for the completeness and accuracy of the data. The first draft of the manuscript was written by the first, second and last authors with input from authors. All the authors participated in reviewing and editing the manuscript and approved the submitted draft.

## Data sharing statement

Data will be shared on request for non-commercial purposes. To submit a request to the Amsterdam UMC COVID-19 Biobank please contact the corresponding author (d.vandebeek@amsterdamumc.nl).

## Declaration of Competing Interest

The authors have declared no potential conflicts of interest.

## Appendix Supplementary materials

Image, application 1

Image, application 1

Image, application 1

Image, application 1

Image, application 1
