# Supplementary material for: Clinical features and prognostic factors in Covid-19: A prospective cohort study
Source: eBioMedicine. 2021 May 14;67:103378. doi: 10.1016/j.ebiom.2021.103378 (PMC8118723; doi:10.1016/j.ebiom.2021.103378)
Supplement: Supplementary file 1 [file mmc1.pdf]

Online supplement

CLINICAL FEATURES AND PROGNOSTIC FACTORS IN COVID-19: A PROSPECTIVE COHORT STUDY

Supplemental methods

Overview of the standard of variation for the Luminex assays.

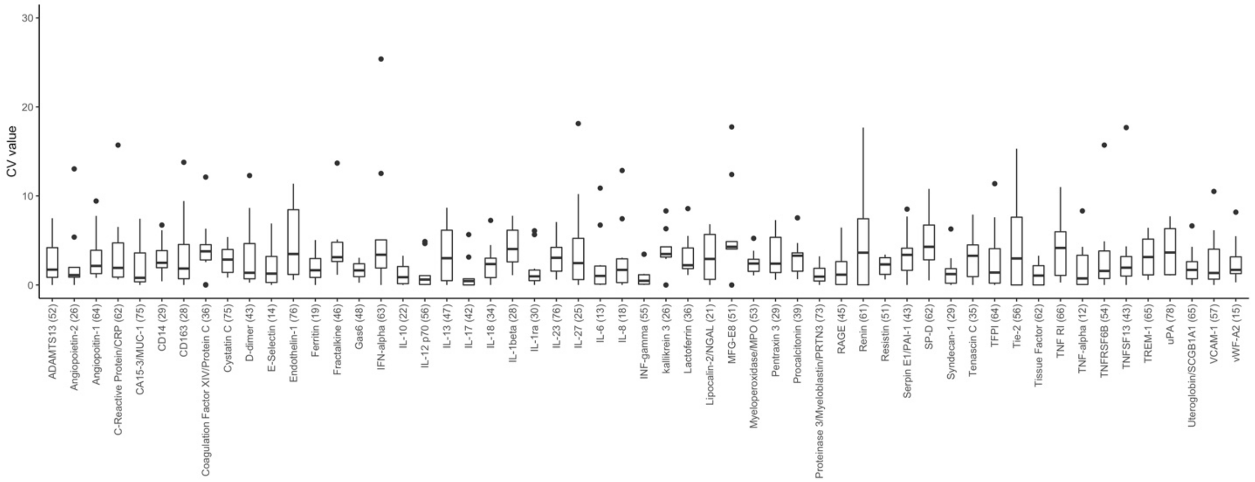

Supplemental Tables

Supplement table 1

Abbreviation	Biomarker	# Ward	# ICU	Producer	Assay	Additional information
C5a	Complement 5a	125	482	BD Biosciences	ELISA	Human C5a ELISA Kit II
C3a	Complement 3a	124	478	Quidel		MicroVue C3a Plus EIA
sC5b-9	Complement complex 5b-9	124	479	Quidel		MicroVue SC5b-9 Plus Enzyme Immunoassay
Bb	Complement factor B	122	479	Quidel		Bb Plus Fragment EIA
MASP-2	Mannan-binding lectin serine protease 2	124	477	MyBiosource		Human Mannan-Binding Lectin Serine Peptidase 2
Tbx2	Thromboxane 2	124	484	R&D Systems		Thromboxane B2 Assay
Thrombomodulin	Thrombomodulin	178	528	R&D Systems	Luminex	3-plex Human magnetic luminex assay
TFF3	Trefoil factor 3	170	510			
uPAR	Urokinase plasminogen activator surface receptor	177	526			
PC	XIV/Protein C	153	467	R&D Systems	Luminex	2-plex Human magnetic luminex assay
Cystatin C	Cystatin C	179	534			
CRP	C-reactive protein	101	308	R&D Systems	Luminex	3-plex Human magnetic luminex assay
CD14	CD14	179	533			
PAI-1	Serpin E1/PAI1 (BR43)	179	533			
ADAMTS13	Structure of von Willebrand factor-cleaving protease	177	531	R&D Systems	Luminex	21-plex Human magnetic luminex assay
ANG-2	Angiopoietin-2	177	530			
ANG-1	Angiopoietin-1	177	530			
CD163	CD163	178	531			
CF	Coagulation factor	153	467			
RAGE	Receptor for advanced glycation endproducts	177	530			
E-Selectin	E-Selectin	177	530			
IL-10	Interleukin-10	133	408			
IL-12 p70	Interleukin-12 p70	28	163			
IL-17	Interleukin-17	93	326			
IL-1ra	Interleukin-1 receptor antagonist	175	502			
IL-23	Interleukin-23	171	517			
IL-6	Interleukin-6	177	529			
L-8	Interleukin-8	177	526			
IFN-gamma	Interferon gamma	173	518			
Resistin	Resistin	178	531			
Syndecan-1	Syndecan-1	178	530			
Tenascin C	Tenascin C	175	478			
TNFa	Tumor necrosis factor alpha	175	523			
TNFSF13	TNF superfamily member 13	177	529			
VCAM-1	vascular cell adhesion molecule 1	175	528			
D-dimer	D-dimer	156	492	R&D Systems	Luminex	20-plex Human magnetic luminex assay
Fractalkine	Fractalkine	71	297			

TNFRSF6B	tumor necrosis factor receptor superfamily 6B	86	285			
IFN-alpha	Interferon alpha	99	332			
IL-13	Interleukin-13	80	292			
IL-18	Interleukin-18	155	491			
IL-1beta	Interleukin-1 beta	20	98			
IL-27	Interleukin-27	150	483			
Kallikrein 3	Kallikrein 3	122	460			
MFG-E8	milk fat globule-epidermal growth factor-factor 8	126	448			
PTX3	Pentraxin 3	156	486			
PCT	Procalcitonin	155	469			
Renin	Renin	148	482			
SP-D	Surfactant protein D	156	492			
TFPI	Tissue factor pathway inhibitor	156	492			
Tie-2	Angiopoietin receptor 2	155	492			
TNF RI	Tumor necrosis factor receptor 1	156	492			
TREM-1	Triggering receptor expressed on myeloid cells-1	155	482			
uPA	Tissue plasminogen activator	155	492			
vWF-A2	von Willebrand Factor	157	491			
Ferritin	Ferritin	50	80	R&D Systems	Luminex	8-plex Human magnetic luminex assay
Gas6	Growth arrest 6	179	533			
MUC-1	Mucin 1	179	529			
Lactoferrin	Lactoferrin	178	515			
NGAL	Neutrophil gelatinase-associated lipocalin	179	533			
MPO	Myeloperoxidase	179	533			
PRTN3	Proteinase 3	156	425			
Uteroglobin	Uteroglobin	179	533			

The following biomarkers were measured for this study.
The third column indicates the number of measurements in the ward cohort of sufficient quality. The fourth column indicates the number of mesaurements in the ICU cohort of sufficient quality. Quality was defined as more than 25 beads counted.

Supplemental table 2: Comparison of patients who were and were not included in analysis

	**Included in analysis N= 217**		**Not included N= 200**		**P-value**
Gender, male	143	(65·9)	111	(55·5)	0·04
Age, years	63·4	(11·7)	60·1	(15·8)	0·02
Do not resuscitate order at hospital admission n (%)	42	(19·4)	41	(20·5)	0·23
Do not intubate order at hospital admission (%)	24	(11·1)	43	(21·5)	0·0004
Hypertension	102	(47)	80	(40)	0·16
Diabetes without complications	53	(24)	37	(18)	0·20
Diabetes with complications	15	(7)	11	(6)	0·69
Chronic Pulmonary Disease	26	(12)	31	(16)	0·33
Asthma	26	(12)	14	(7)	0·11
Chronic Kidney Disease	18	(8)	13	(7)	0·58
Liver disease	7	(3)	4	(2)	0·54
Chronic Neurological Disease	31	(14)	27	(14)	0·89
Active Solid Malignancy	12	(6)	11	(6)	1·0
Active Heamatological Disease	9	(4)	10	(5)	0·80
Usage of immunosuppressive agents prior to admission	18	(8)	10	(5)	0·51
Rheumatological disorders	18	(8)	23	(12)	0·33
Pulmonary embolism	41	(19)	7	(4)	0·0004
Renal replacement therapy	24	(11)	7	(4)	0·0001
Mortality	57	(26)	24	(12)	0·007

Supplemental table 3: Baseline demographics non-COVID outpatient clinic cohort

**Non- covid outpatient clinic cohort**	N=26
Male	18 (69·2%)
Age (mean (SD))	63·62 (15·50)
BMI(median [IQR])	26·5 [22·9, 29·3]
Medical history	
Malignancy (%)	10 (38·4%)
Hematological	1 (3·8%)
Solid	9 (34·6%)
Immunosuppressant use	4 (15·4%)
Statins = TRUE (%)	11 (42·3%)
NSAIDs use	2 (7·7%)
COPD	0 (0%)
Hypertension	11 (42·3%)
Diabetes	5 (19·2%)

Supplemental table 4: Baseline demographics non-COVID ICU cohort

**ICU non-COVID cohort**	**N= 25**
Male	17 (68%)
Age	60·1 (17·6)
BMI (median [IQR])	26·34[24·5, 30·6]
Mechanically ventilated in first 24 hours	22 (88·0%)
Main reason of admission	
Respiratory insufficiency	6 (24%)
Pneumonia	4 (16%)
Asthma renale	1 (4%)
Asthma cardiale	1 (4%)
Cardiac arrest	1 (4%)
Trauma	4 (16%)
Postsurgery	7 (28%)
Cardiothoracic	5 (20%)
Other	2 (8%)
GI bleeding	2 (8%)
Other	2 (8%)
Medical history	
Chronic renal insufficiency	6 (24%)
Neoplasm	1 (4%)
Hematologic malignancy	1 (4%)
Liver cirrhosis	3 (12%)
Imune deficiency	6 (24%)
Outcome	
90-day mortality	5(20%)

Supplemental table 5: Hazard ratios for joint model analysis, stratified per biomarker and cohort

	Cytokine	Cohort	HR	Lower CI	Higher CI	P-value	n	events	measurements
1	ADAMTS13	IC	1·034316748	0·748931902	1·428449146	0·837696554	115	43	531
2	Angiopoietin-2	IC	3·00263619	1·894397764	4·759203299	2·89E-06	115	43	530
3	Angiopoitin-1	IC	0·975313228	0·586331875	1·62235064	0·923299645	115	43	530
4	Bb	IC	2·075579279	1·320350932	3·262791156	0·001555476	112	42	479
5	C-Reactive Protein/CRP	IC	2·412498878	0·268696586	21·66068022	0·431611577	104	38	308
6	C3a	IC	13·2367175	2·662335173	65·81090616	0·001595714	112	42	478
7	C5a	IC	3·384288302	1·853079269	6·180743318	7·27E-05	112	42	482
8	C5b-9	IC	0·526234032	0·326980079	0·846908647	0·008183139	112	42	479
9	CA15-3/MUC-1	IC	0·814437047	0·688889326	0·962865411	0·0162601	115	43	529
10	CD14	IC	9·90279511	3·308505393	29·64037816	4·15E-05	115	43	533
11	CD163	IC	0·978310981	0·614183351	1·55831703	0·92644354	115	43	531
12	Coagulation Factor XIV/Protein C	IC	1·456842019	0·484036425	4·384770564	0·503297014	112	40	467
13	Cystatin C	IC	1·478628809	0·837759037	2·609751798	0·17724372	115	43	534
14	D-dimer	IC	3·072895999	1·297402967	7·278147235	0·010715679	111	40	492
15	E-Selectin	IC	1·626125493	0·72149977	3·664982625	0·240921612	115	43	530
16	Fractalkine	IC	0·528254851	0·283967985	0·982692425	0·043891081	102	40	297
17	Gas6	IC	12·81646962	3·577221114	45·91885386	8·94E-05	115	43	533
18	IFN-alpha	IC	171·3632551	1·425411178	20601·32939	0·035286037	101	36	332
19	IL-10	IC	2·228716495	1·671853497	2·971060097	4·66E-08	113	42	408
20	IL-12 p70	IC	0·000247484	4·48E-07	0·136631973	0·009939939	71	30	163
21	IL-13	IC	0·564988044	0·308999233	1·033049459	0·063682795	102	39	292
22	IL-17	IC	732·9043725	3·288524606	163340·368	0·016776955	105	39	326
23	IL-18	IC	1·660860183	1·122103682	2·458290255	0·011217975	111	40	491
24	IL-1ra	IC	3·166738767	1·951745362	5·138085434	3·04E-06	115	43	502
25	IL-23	IC	3·996817273	1·367368755	11·68269222	0·011349602	115	43	517
26	IL-27	IC	2·053954357	1·149453626	3·670203311	0·015086175	110	40	483
27	IL-6	IC	2·726655804	1·86897934	3·977920846	1·93E-07	115	43	529
28	IL-8	IC	3·115890862	1·917128957	5·064226812	4·51E-06	114	42	526
29	INF-gamma	IC	2·638356792	1·322564809	5·263202616	0·00589657	115	43	518
30	kallikrein 3	IC	1·129533584	0·980175866	1·301650206	0·092319461	109	38	460
31	Lactoferrin	IC	0·686375685	0·565400337	0·83323541	0·000142244	115	43	515
32	Lipocalin-2/NGAL	IC	1·058677123	0·764307836	1·466421247	0·73157902	115	43	533
33	MASP2	IC	0·40759177	0·093676985	1·773445751	0·231573404	112	42	477
34	MFG-E8	IC	2·561874245	1·342050984	4·890424975	0·004346279	110	40	448
35	Myeloperoxidase/MPO	IC	0·490945968	0·269336043	0·894896729	0·020204563	115	43	533
36	Pentraxin 3	IC	2·325490981	1·482091388	3·648835926	0·000240757	111	40	486
37	Procalcitonin	IC	2·283047313	1·655216907	3·14901631	4·87E-07	111	40	469
38	Proteinase 3/Myeloblastin/PRTN3	IC	1·997657676	0·669262906	5·962733261	0·214885732	110	41	425
39	RAGE	IC	1·953253639	1·419077908	2·688506216	4E-05	115	43	530

**40**	Renin	IC	1·254700699	0·961108765	1·637976785	0·095250046	111	40	482
**41**	Resistin	IC	1·968424767	0·927909367	4·175726853	0·077563518	115	43	531
**42**	Serpin E1/PAI-1	IC	3·092405435	1·428479162	6·694512338	0·00417027	115	43	533
**43**	SP-D	IC	1·032167878	0·735463668	1·448569896	0·854717755	111	40	492
**44**	Syndecan-1	IC	0·562866594	0·399975521	0·792095482	0·000976706	115	43	530
**45**	TBX2	IC	0·06469258	0·001334395	3·136350492	0·166740961	112	42	484
**46**	Tenascin C	IC	0·355712024	0·180639343	0·700462271	0·002791913	114	42	478
**47**	TFF3	IC	2·026176418	0·222494459	18·45165446	0·530953136	113	41	510
**48**	TFPI	IC	0·777240039	0·520327056	1·16100455	0·218377106	111	40	492
**49**	Thrombomodulin	IC	1·321519198	0·755928571	2·310288376	0·327975786	115	43	528
**50**	Tie-2	IC	0·461736385	0·279317059	0·763292048	0·002584455	111	40	489
**51**	Tissue Factor	IC	3·696786448	1·950180987	7·007672689	6·15E-05	115	43	527
**52**	TNF RI	IC	1·57260913	1·03942666	2·379291942	0·032109696	111	40	492
**53**	TNF-alpha	IC	3·855008655	2·108474636	7·048266779	1·17E-05	115	43	523
**54**	TNFRSF6B	IC	0·111743181	0·006272056	1·990820532	0·135850816	95	39	285
**55**	TNFSF13	IC	1·284477671	0·259929822	6·347416671	0·758749224	115	43	529
**56**	TREM-1	IC	1·412553127	0·834428902	2·391223903	0·198427921	111	40	488
**57**	uPA	IC	1·096015376	0·469380785	2·559222155	0·832185701	111	40	492
**58**	uPAR	IC	3·096659373	2·068415802	4·636059761	4·02E-08	115	43	526
**59**	Uteroglobin/SCGB1A1	IC	0·92730161	0·691330163	1·243817095	0·614432653	115	43	533
**60**	VCAM-1	IC	2·581969602	1·599805697	4·167110443	0·000102745	115	43	528
**61**	vWF-A2	IC	3·044203096	1·283545909	7·219977427	0·011519349	111	40	491
**62**	ADAMTS13	Ward	1·188132801	0·740350416	1·906745135	0·475046526	98	24	177
**63**	Angiopoietin-2	Ward	1·32744468	0·910391906	1·935550356	0·140994161	98	24	177
**64**	Angiopoitin-1	Ward	0·362758151	0·176656281	0·744912525	0·005741571	98	24	177
**65**	Bb	Ward	81·69462079	0·17073081	39090·84161	0·161953475	66	13	122
**66**	C-Reactive Protein/CRP	Ward	1·53	0·3	7·86	0·613747	65	11	101
**67**	C3a	Ward	1·47	0·81	2·66	0·2048353	65	12	124
**68**	C5a	Ward	11·31180925	0·835006629	153·2407337	0·068092951	66	13	125
**69**	C5b-9	Ward	0·367055411	0·147904882	0·91092108	0·030681027	66	13	125
**70**	CA15-3/MUC-1	Ward	0·725496533	0·559419377	0·940877705	0·01554246	99	24	179
**71**	CD14	Ward	3·641910246	1·548454661	8·565643266	0·003055745	99	24	179
**72**	CD163	Ward	1·036543737	0·64352711	1·66958455	0·882675645	98	24	178
**73**	Coagulation Factor XIV/Protein C	Ward	0·339563939	0·154643554	0·745609279	0·007112841	89	20	153
**74**	Cystatin C	Ward	0·427636227	0·179194251	1·020527953	0·055593201	99	24	179
**75**	D-dimer	Ward	0·533841361	0·282847438	1·007562951	0·052775799	92	20	156
**76**	E-Selectin	Ward	1·202564486	0·471078545	3·069894306	0·699670258	98	24	177
**77**	Fractalkine	Ward	5·115557389	0·544739972	48·03930086	0·153170781	56	9	71
**78**	Gas6	Ward	3·160886126	0·900649262	11·0933318	0·072392288	99	24	179
**79**	IFN-alpha	Ward	1·851842725	0·379658055	9·032658281	0·445984657	72	15	99
**80**	IL-10	Ward	1·836970458	1·448283286	2·329972661	5·34E-07	80	21	133
**81**	IL-13	Ward	0·629514228	0·333606482	1·187891077	0·153137411	62	14	80

**82**	IL-17	Ward	6·463276868	0·942272552	44·33318976	0·057501793	65	16	93
**83**	IL-18	Ward	1·449146601	0·717641129	2·926289735	0·300832705	92	20	155
**84**	IL-1ra	Ward	5·784966844	1·80881828	18·50149446	0·003084546	98	24	175
**85**	IL-23	Ward	0·313971862	0·12136567	0·812242295	0·016902411	95	24	171
**86**	IL-27	Ward	3·84738756	1·271019106	11·64608067	0·017107403	91	20	150
**87**	IL-6	Ward	3·02176861	1·705270582	5·354625611	0·000151581	98	24	177
**88**	IL-8	Ward	4·423536264	2·373787013	8·243230318	2·84E-06	98	24	177
**89**	INF-gamma	Ward	11·3692727	0·948229173	136·3176385	0·0551039	97	24	173
**90**	kallikrein 3	Ward	0·878819333	0·625101273	1·235517275	0·457356843	75	16	122
**91**	Lactoferrin	Ward	0·96864751	0·63408868	1·479726778	0·882854162	98	24	178
**92**	Lipocalin-2/NGAL	Ward	0·33259801	0·171776062	0·643986333	0·001092987	99	24	179
**93**	MASP2	Ward	0·171884261	0·03899593	0·757622631	0·019978102	65	13	124
**94**	MFG-E8	Ward	4·987256345	0·888369727	27·99816911	0·067921529	81	20	126
**95**	Myeloperoxidase/MPO	Ward	0·365929932	0·174280238	0·768329884	0·007899344	99	24	179
**96**	Pentraxin 3	Ward	3·574348647	1·787392782	7·147823567	0·000315185	92	20	156
**97**	Procalcitonin	Ward	2·053439817	1·193055507	3·534299164	0·009399934	92	20	155
**98**	Proteinase 3/Myeloblastin/PRTN3	Ward	0·368809531	0·140491371	0·96817669	0·042797831	93	20	156
**99**	RAGE	Ward	2·197356364	1·5185611	3·17957242	2·97E-05	98	24	177
**100**	Renin	Ward	1·502925648	1·087268364	2·077486643	0·013642513	91	20	148
**101**	Resistin	Ward	1·298300162	0·611769613	2·75525831	0·496504625	98	24	178
**102**	Serpin E1/PAI-1	Ward	1·06	0·52	2·16	0·8829481	99	24	179
**103**	SP-D	Ward	0·767655727	0·635631437	0·927102219	0·006030788	92	20	156
**104**	Syndecan-1	Ward	0·961845335	0·910832905	1·015714784	0·161759176	98	24	178
**105**	TBX2	Ward	58·69832881	0·121049199	28463·58192	0·196792205	65	13	124
**106**	Tenascin C	Ward	3·148727542	0·580569789	17·07716337	0·183629802	97	23	175
**107**	TFF3	Ward	2·53370752	1·278359101	5·021807873	0·007731135	98	24	170
**108**	TFPI	Ward	0·659021601	0·459944209	0·944265546	0·023054513	92	20	156
**109**	Thrombomodulin	Ward	2·092339549	1·143894528	3·827175219	0·016558339	99	24	178
**110**	Tie-2	Ward	1·716819586	1·006380961	2·928781052	0·047329306	92	20	155
**111**	Tissue Factor	Ward	1·654680954	1·061318729	2·579780216	0·026239085	98	24	176
**112**	TNF RI	Ward	1·667274839	0·76742678	3·62224184	0·196595118	92	20	156
**113**	TNF-alpha	Ward	4·761282842	1·276627181	17·75758392	0·020144121	96	24	175
**114**	TNFRSF6B	Ward	0·09568063	0·0073125	1·251936152	0·07365743	58	11	86
**115**	TNFSF13	Ward	6·096031491	1·702934558	21·82209513	0·005466444	98	24	177
**116**	TREM-1	Ward	0·880706672	0·521288273	1·487937256	0·6349502	92	20	155
**117**	uPA	Ward	6·26957971	1·573059358	24·98801431	0·009263519	92	20	155
**118**	uPAR	Ward	1·939478967	1·31758236	2·854909703	0·000784578	99	24	177
**119**	Uteroglobin/SCGB1A1	Ward	0·724604374	0·497161092	1·056099335	0·093735634	99	24	179
**120**	VCAM-1	Ward	3·83187247	1·766651841	8·311341423	0·000672384	97	23	175
**121**	vWF-A2	Ward	0·604910497	0·424290077	0·862421088	0·005470015	93	21	157

Supplemental table 6: Pathways associated with worse outcomes in the ward cohort

ID	Description	GeneRatio	BgRatio	pvalue	p·adjust	qvalue	geneID	Count
GO:0032642	regulation of chemokine production	3/5	68/18866	4·46e-07	3·43e-04	3·98e-05	IL10/IL6/AGER	3
GO:0032602	chemokine production	3/5	75/18866	6·00e-07	4·62e-04	3·98e-05	IL10/IL6/AGER	3
GO:0042116	macrophage activation	3/5	101/18866	1·48e-06	1·14e-03	6·27e-05	IL10/IL6/AGER	3
GO:0032652	regulation of interleukin-1 production	3/5	111/18866	1·97e-06	1·51e-03	6·27e-05	IL10/IL6/AGER	3
GO:0032612	interleukin-1 production	3/5	121/18866	2·55e-06	1·96e-03	6·27e-05	IL10/IL6/AGER	3
GO:0002687	positive regulation of leukocyte migration	3/5	142/18866	4·13e-06	3·18e-03	6·27e-05	IL6/CXCL8/AGER	3
GO:0050921	positive regulation of chemotaxis	3/5	144/18866	4·31e-06	3·32e-03	6·27e-05	IL6/CXCL8/AGER	3
GO:0050930	induction of positive chemotaxis	2/5	14/18866	5·11e-06	3·93e-03	6·27e-05	CXCL8/AGER	2
GO:0002822	regulation of adaptive immune response based on somatic recombination of immune receptors built from immunoglobulin superfamily domains	3/5	153/18866	5·17e-06	3·98e-03	6·27e-05	IL10/IL6/AGER	3
GO:0002706	regulation of lymphocyte mediated immunity	3/5	154/18866	5·27e-06	4·06e-03	6·27e-05	IL10/IL6/AGER	3
GO:0032675	regulation of interleukin-6 production	3/5	159/18866	5·80e-06	4·47e-03	6·27e-05	IL10/IL6/AGER	3
GO:0042129	regulation of T cell proliferation	3/5	167/18866	6·72e-06	5·18e-03	6·27e-05	IL10/IL6/AGER	3
GO:0034116	positive regulation of heterotypic cell-cell adhesion	2/5	16/18866	6·73e-06	5·18e-03	6·27e-05	IL10/AGER	2
GO:0002819	regulation of adaptive immune response	3/5	168/18866	6·85e-06	5·27e-03	6·27e-05	IL10/IL6/AGER	3
GO:0032635	interleukin-6 production	3/5	170/18866	7·09e-06	5·46e-03	6·27e-05	IL10/IL6/AGER	3
GO:0042098	T cell proliferation	3/5	195/18866	1·07e-05	8·25e-03	8·33e-05	IL10/IL6/AGER	3
GO:0034104	negative regulation of tissue remodeling	2/5	22/18866	1·30e-05	9·97e-03	8·33e-05	IL6/AGER	2

Supplemental table 7: Pathways associated with worse outcomes in the ICU cohort

ID	Description	GeneRatio	BgRatio	pvalue	p·adjust	qvalue	geneID	Count
GO:0050920	regulation of chemotaxis	7/15	229/18866	2·10e-10	2·75e-07	7·53e-08	ANGPT2/C5/GAS6/IL6/CXCL8/AGER/F3	7
GO:0034113	heterotypic cell-cell adhesion	5/15	62/18866	9·52e-10	1·25e-06	1·71e-07	IL10/IL1RN/AGER/TNF/VCAM1	5
GO:0034114	regulation of heterotypic cell-cell adhesion	4/15	26/18866	3·83e-09	5·02e-06	3·43e-07	IL10/IL1RN/AGER/TNF	4
GO:0050926	regulation of positive chemotaxis	4/15	26/18866	3·83e-09	5·02e-06	3·43e-07	ANGPT2/CXCL8/AGER/F3	4
GO:0032677	regulation of interleukin-8 production	5/15	93/18866	7·54e-09	9·89e-06	4·42e-07	CD14/IL10/IL6/F3/TNF	5
GO:0071222	cellular response to lipopolysaccharide	6/15	208/18866	7·70e-09	1·01e-05	4·42e-07	CD14/IL10/IL6/CXCL8/LTF/TNF	6
GO:0002685	regulation of leukocyte migration	6/15	212/18866	8·63e-09	1·13e-05	4·42e-07	C5/GAS6/IL6/CXCL8/AGER/TNF	6
GO:0071219	cellular response to molecule of bacterial origin	6/15	222/18866	1·14e-08	1·49e-05	4·57e-07	CD14/IL10/IL6/CXCL8/LTF/TNF	6
GO:0032637	interleukin-8 production	5/15	101/18866	1·15e-08	1·50e-05	4·57e-07	CD14/IL10/IL6/F3/TNF	5
GO:0032102	negative regulation of response to external stimulus	7/15	433/18866	1·75e-08	2·30e-05	5·23e-07	ANGPT2/C5/IL10/LTF/AGER/TNF/PLAUR	7
GO:0052548	regulation of endopeptidase activity	7/15	434/18866	1·78e-08	2·34e-05	5·23e-07	C5/GAS6/LTF/AGER/F3/TNF/PLAUR	7
GO:0032652	regulation of interleukin-1 production	5/15	111/18866	1·84e-08	2·42e-05	5·23e-07	GAS6/IL10/IL6/AGER/TNF	5
GO:0022407	regulation of cell-cell adhesion	7/15	439/18866	1·93e-08	2·53e-05	5·23e-07	IL10/IL1RN/IL6/AGER/TNF/PLAUR/VCAM1	7
GO:0071216	cellular response to biotic stimulus	6/15	246/18866	2·10e-08	2·75e-05	5·23e-07	CD14/IL10/IL6/CXCL8/LTF/TNF	6
GO:0001819	positive regulation of cytokine production	7/15	447/18866	2·18e-08	2·86e-05	5·23e-07	C5/CD14/IL10/IL6/AGER/F3/TNF	7
GO:0032612	interleukin-1 production	5/15	121/18866	2·85e-08	3·73e-05	6·14e-07	GAS6/IL10/IL6/AGER/TNF	5
GO:0052547	regulation of peptidase activity	7/15	466/18866	2·91e-08	3·81e-05	6·14e-07	C5/GAS6/LTF/AGER/F3/TNF/PLAUR	7
GO:0022409	positive regulation of cell-cell adhesion	6/15	279/18866	4·44e-08	5·82e-05	8·85e-07	IL10/IL6/AGER/TNF/PLAUR/VCAM1	6
GO:0002687	positive regulation of leukocyte migration	5/15	142/18866	6·36e-08	8·34e-05	1·20e-06	GAS6/IL6/CXCL8/AGER/TNF	5
GO:0050921	positive regulation of chemotaxis	5/15	144/18866	6·82e-08	8·95e-05	1·22e-06	GAS6/IL6/CXCL8/AGER/F3	5
GO:0032757	positive regulation of interleukin-8 production	4/15	54/18866	7·99e-08	1·05e-04	1·37e-06	CD14/IL6/F3/TNF	4
GO:0032675	regulation of interleukin-6 production	5/15	159/18866	1·12e-07	1·47e-04	1·83e-06	GAS6/IL10/IL6/AGER/TNF	5
GO:0032496	response to lipopolysaccharide	6/15	334/18866	1·29e-07	1·69e-04	2·01e-06	CD14/IL10/IL6/CXCL8/LTF/TNF	6
GO:0032635	interleukin-6 production	5/15	170/18866	1·56e-07	2·05e-04	2·34e-06	GAS6/IL10/IL6/AGER/TNF	5
GO:0002237	response to molecule of bacterial origin	6/15	356/18866	1·88e-07	2·46e-04	2·66e-06	CD14/IL10/IL6/CXCL8/LTF/TNF	6

GO:0050918	positive chemotaxis	4/15	67/18866	1·93e-07	2·52e-04	2·66e-06	ANGPT2/CXCL8/AGER/F3	4
GO:0032642	regulation of chemokine production	4/15	68/18866	2·04e-07	2·68e-04	2·72e-06	IL10/IL6/AGER/TNF	4
GO:1903034	regulation of response to wounding	5/15	183/18866	2·26e-07	2·96e-04	2·77e-06	IL10/AGER/F3/TNF/PLAUR	5
GO:0034116	positive regulation of heterotypic cell-cell adhesion	3/15	16/18866	2·26e-07	2·97e-04	2·77e-06	IL10/AGER/TNF	3
GO:0045861	negative regulation of proteolysis	6/15	369/18866	2·32e-07	3·04e-04	2·77e-06	C5/GAS6/IL10/LTF/TNF/PLAUR	6
GO:0031100	animal organ regeneration	4/15	75/18866	3·04e-07	3·99e-04	3·41e-06	ANGPT2/GAS6/IL10/IL6	4
GO:0032602	chemokine production	4/15	75/18866	3·04e-07	3·99e-04	3·41e-06	IL10/IL6/AGER/TNF	4
GO:0045765	regulation of angiogenesis	6/15	403/18866	3·89e-07	5·10e-04	4·23e-06	ANGPT2/IL10/IL6/CXCL8/F3/TNF	6
GO:0045785	positive regulation of cell adhesion	6/15	428/18866	5·54e-07	7·26e-04	5·85e-06	IL10/IL6/AGER/TNF/PLAUR/VCAM1	6
GO:1901342	regulation of vasculature development	6/15	444/18866	6·87e-07	9·00e-04	7·04e-06	ANGPT2/IL10/IL6/CXCL8/F3/TNF	6
GO:2000117	negative regulation of cysteine-type endopeptidase activity	4/15	93/18866	7·25e-07	9·50e-04	7·10e-06	GAS6/LTF/TNF/PLAUR	4
GO:0030595	leukocyte chemotaxis	5/15	232/18866	7·32e-07	9·59e-04	7·10e-06	C5/GAS6/IL10/IL6/CXCL8	5
GO:0051090	regulation of DNA-binding transcription factor activity	6/15	455/18866	7·92e-07	1·04e-03	7·48e-06	GAS6/IL10/IL6/LTF/AGER/TNF	6
GO:2000116	regulation of cysteine-type endopeptidase activity	5/15	239/18866	8·47e-07	1·11e-03	7·80e-06	GAS6/LTF/F3/TNF/PLAUR	5
GO:0050927	positive regulation of positive chemotaxis	3/15	25/18866	9·25e-07	1·21e-03	8·30e-06	CXCL8/AGER/F3	3
GO:0051346	negative regulation of hydrolase activity	6/15	473/18866	9·94e-07	1·30e-03	8·63e-06	C5/GAS6/LTF/PTX3/TNF/PLAUR	6
GO:0042116	macrophage activation	4/15	101/18866	1·01e-06	1·32e-03	8·63e-06	IL10/IL6/AGER/TNF	4
GO:0002367	cytokine production involved in immune response	4/15	104/18866	1·14e-06	1·49e-03	9·26e-06	GAS6/IL10/IL6/TNF	4
GO:0032649	regulation of interferon-gamma production	4/15	104/18866	1·14e-06	1·49e-03	9·26e-06	CD14/GAS6/IL10/TNF	4
GO:0048143	astrocyte activation	3/15	27/18866	1·18e-06	1·54e-03	9·38e-06	IL6/AGER/TNF	3
GO:0010951	negative regulation of endopeptidase activity	5/15	258/18866	1·24e-06	1·62e-03	9·64e-06	C5/GAS6/LTF/TNF/PLAUR	5
GO:0032680	regulation of tumor necrosis factor production	4/15	110/18866	1·42e-06	1·86e-03	1·09e-05	CD14/GAS6/IL10/LTF	4
GO:0051091	positive regulation of DNA-binding transcription factor activity	5/15	270/18866	1·55e-06	2·03e-03	1·15e-05	IL10/IL6/LTF/AGER/TNF	5
GO:1903555	regulation of tumor necrosis factor superfamily cytokine production	4/15	113/18866	1·58e-06	2·08e-03	1·15e-05	CD14/GAS6/IL10/LTF	4
GO:0010466	negative regulation of peptidase activity	5/15	272/18866	1·60e-06	2·10e-03	1·15e-05	C5/GAS6/LTF/TNF/PLAUR	5
GO:0032609	interferon-gamma production	4/15	115/18866	1·70e-06	2·23e-03	1·20e-05	CD14/GAS6/IL10/TNF	4
GO:0032640	tumor necrosis factor production	4/15	116/18866	1·76e-06	2·30e-03	1·21e-05	CD14/GAS6/IL10/LTF	4

GO:1904019	epithelial cell apoptotic process	4/15	117/18866	1·82e-06	2·38e-03	1·23e-05	GAS6/IL10/IL6/TNF	4
GO:0071706	tumor necrosis factor superfamily cytokine production	4/15	119/18866	1·95e-06	2·55e-03	1·29e-05	CD14/GAS6/IL10/LTF	4
GO:0002688	regulation of leukocyte chemotaxis	4/15	124/18866	2·29e-06	3·01e-03	1·50e-05	C5/GAS6/IL6/CXCL8	4
GO:0050715	positive regulation of cytokine secretion	3/15	34/18866	2·40e-06	3·14e-03	1·54e-05	CD14/IL10/TNF	3
GO:0019079	viral genome replication	4/15	127/18866	2·52e-06	3·31e-03	1·59e-05	GAS6/CXCL8/LTF/TNF	4
GO:0060326	cell chemotaxis	5/15	311/18866	3·09e-06	4·05e-03	1·91e-05	C5/GAS6/IL10/IL6/CXCL8	5
GO:1903037	regulation of leukocyte cell-cell adhesion	5/15	329/18866	4·07e-06	5·34e-03	2·48e-05	IL10/IL6/AGER/TNF/VCAM1	5
GO:0051384	response to glucocorticoid	4/15	147/18866	4·52e-06	5·92e-03	2·70e-05	IL10/IL1RN/IL6/TNF	4
GO:0019058	viral life cycle	5/15	341/18866	4·85e-06	6·36e-03	2·85e-05	GAS6/CXCL8/LTF/PTX3/TNF	5
GO:0061041	regulation of wound healing	4/15	151/18866	5·03e-06	6·59e-03	2·91e-05	AGER/F3/TNF/PLAUR	4
GO:0002822	regulation of adaptive immune response based on somatic recombination of immune receptors built from immunoglobulin superfamily domains	4/15	153/18866	5·30e-06	6·94e-03	3·02e-05	IL10/IL6/AGER/TNF	4
GO:0002706	regulation of lymphocyte mediated immunity	4/15	154/18866	5·43e-06	7·12e-03	3·05e-05	IL10/IL6/AGER/TNF	4
GO:0014002	astrocyte development	3/15	45/18866	5·66e-06	7·41e-03	3·08e-05	IL6/AGER/TNF	3
GO:0032722	positive regulation of chemokine production	3/15	45/18866	5·66e-06	7·41e-03	3·08e-05	IL6/AGER/TNF	3
GO:0051222	positive regulation of protein transport	5/15	354/18866	5·82e-06	7·63e-03	3·10e-05	CD14/GAS6/IL10/IL6/TNF	5
GO:1903900	regulation of viral life cycle	4/15	157/18866	5·87e-06	7·69e-03	3·10e-05	CXCL8/LTF/PTX3/TNF	4
GO:0001818	negative regulation of cytokine production	5/15	360/18866	6·31e-06	8·28e-03	3·28e-05	GAS6/IL10/LTF/AGER/TNF	5
GO:0007159	leukocyte cell-cell adhesion	5/15	364/18866	6·66e-06	8·73e-03	3·42e-05	IL10/IL6/AGER/TNF/VCAM1	5
GO:0002449	lymphocyte mediated immunity	5/15	366/18866	6·84e-06	8·97e-03	3·46e-05	C5/IL10/IL6/AGER/TNF	5
GO:0031960	response to corticosteroid	4/15	164/18866	6·97e-06	9·14e-03	3·48e-05	IL10/IL1RN/IL6/TNF	4
GO:0002460	adaptive immune response based on somatic recombination of immune receptors built from immunoglobulin superfamily domains	5/15	370/18866	7·21e-06	9·46e-03	3·50e-05	C5/IL10/IL6/AGER/TNF	5
GO:1904951	positive regulation of establishment of protein localization	5/15	370/18866	7·21e-06	9·46e-03	3·50e-05	CD14/GAS6/IL10/IL6/TNF	5
GO:0042129	regulation of T cell proliferation	4/15	167/18866	7·49e-06	9·82e-03	3·59e-05	IL10/IL6/AGER/VCAM1	4

Supplemental figures

Supplemental Figure 1. Flow chart

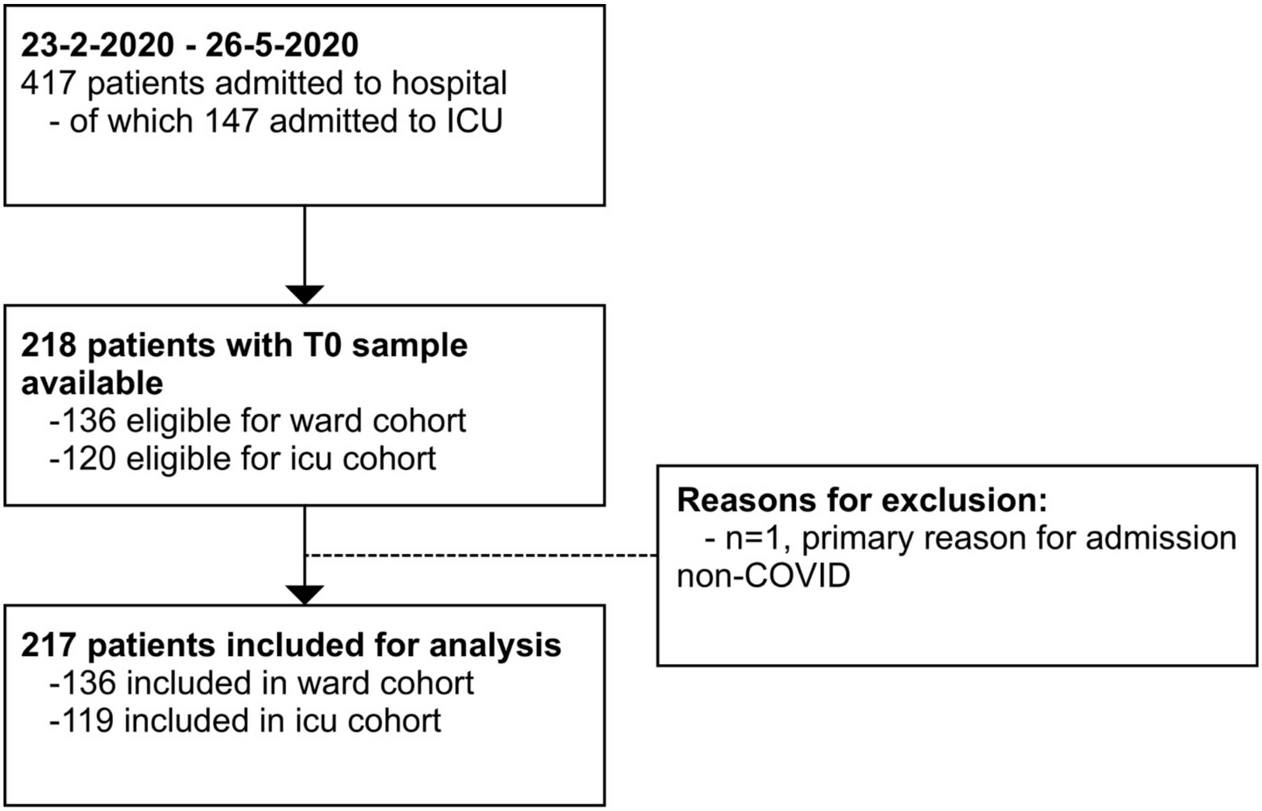

Supplemental figure 2 Kaplan- Meier curve ward (panel A) and ICU (panel B) cohort

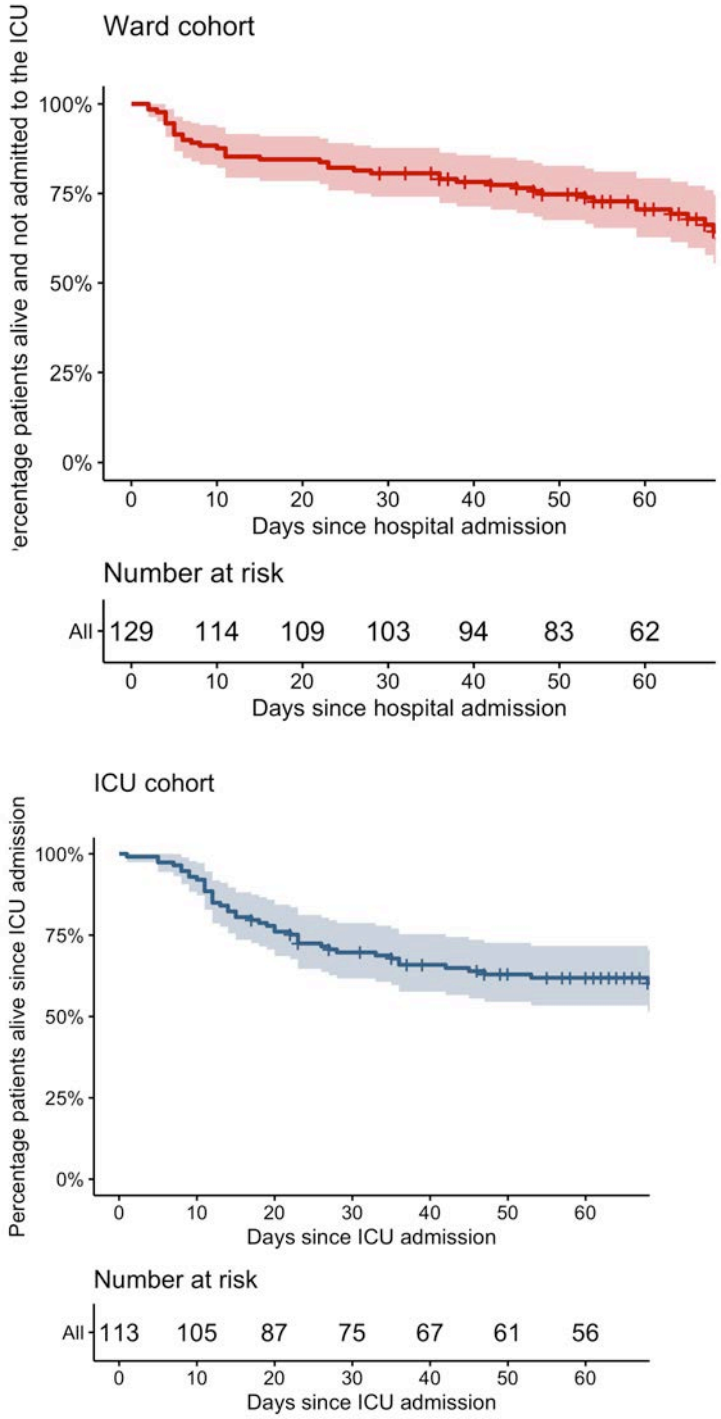

Supplemental figure 3. Vulcanoplot.

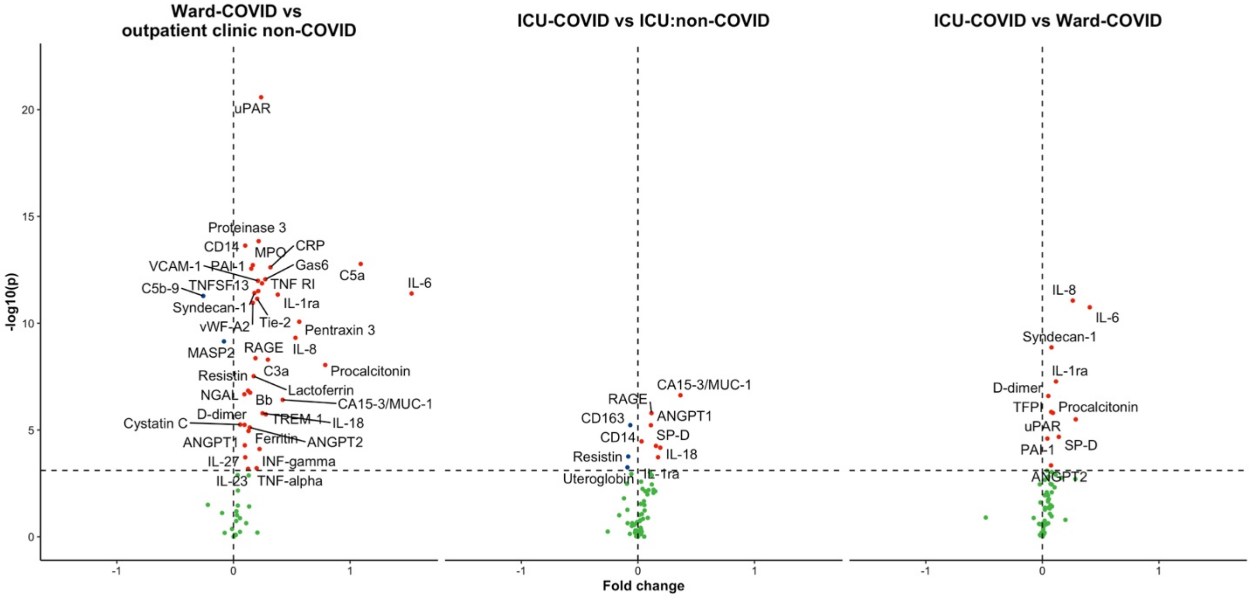

Differences in baseline biomarker concentrations shown in volcano plot. X-axis shows foldchange, Y-axis -log10 of the p-value. Each dot represents a biomarker. Red dots indicate significant biomarkers, after adjustment for multiple testing.

Supplemental figure 4: Hazard ratios for unfavorable outcome for all biomarkers for the ward cohort

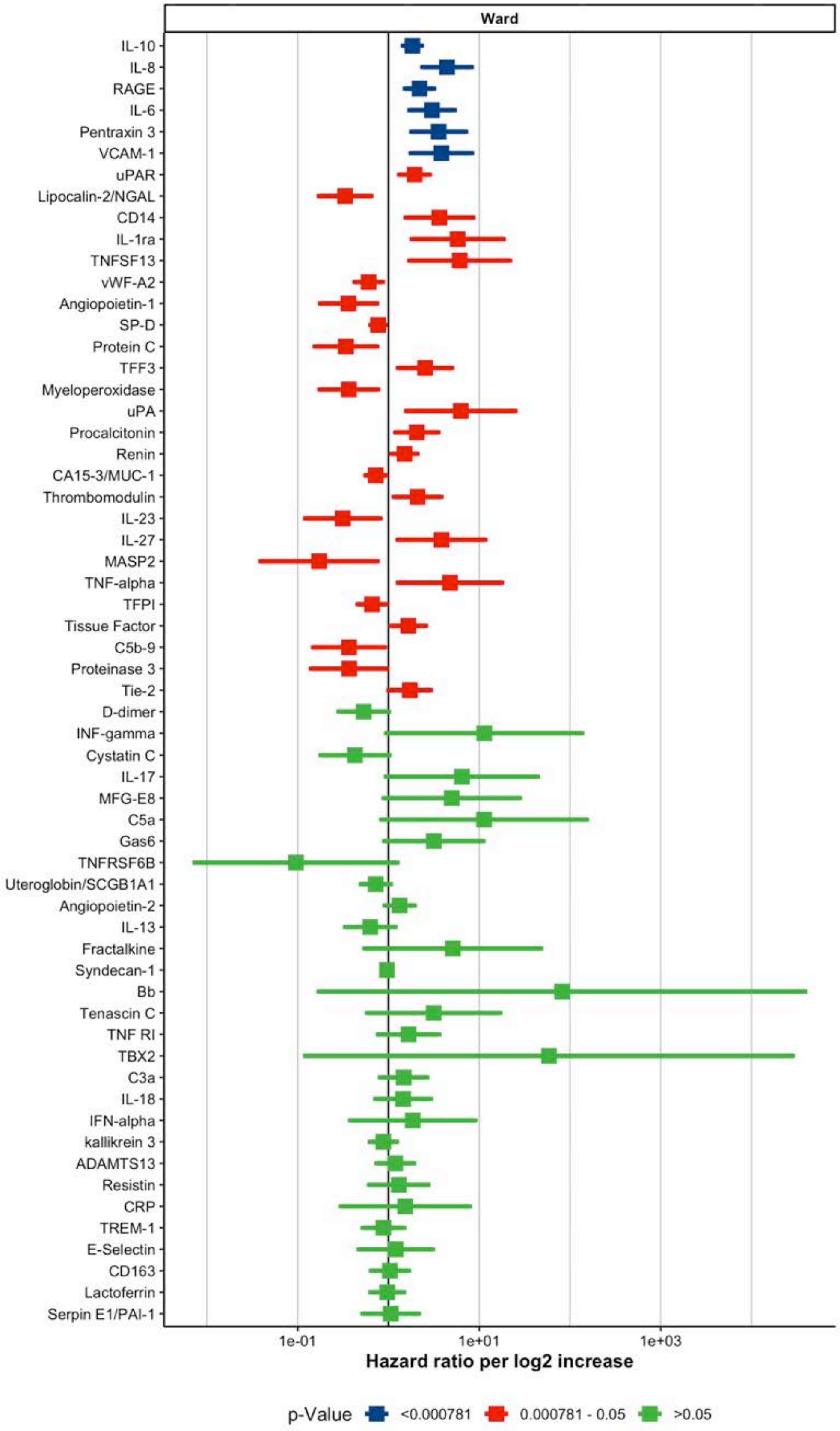

Supplemental figure 5: Hazard ratios for unfavorable outcome for all biomarkers for the ICU cohort

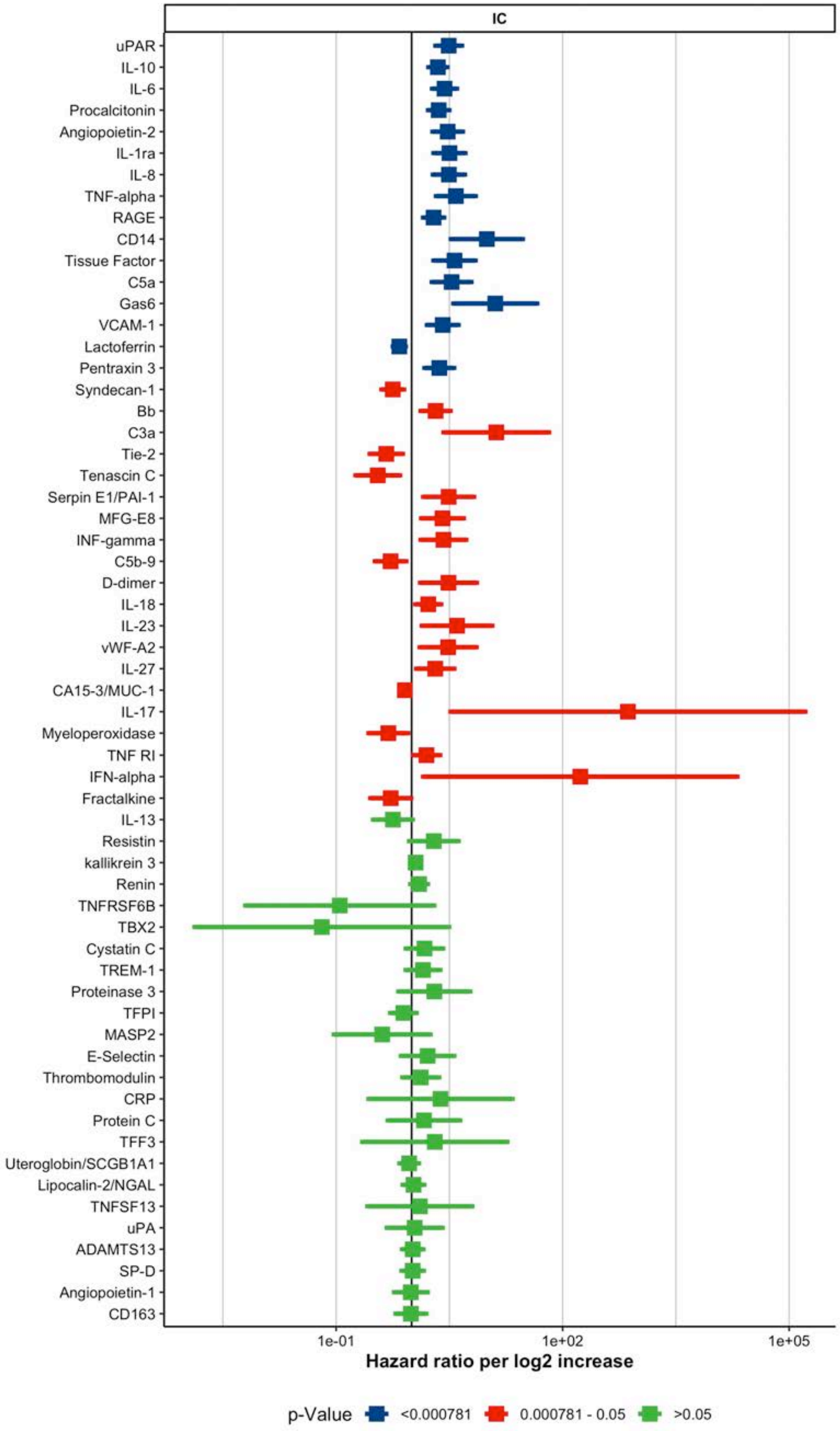

Supplemental figure 6. Absolute biomarker concentrations during stay on the ward, stratified for favourable and non-favourable outcoes.

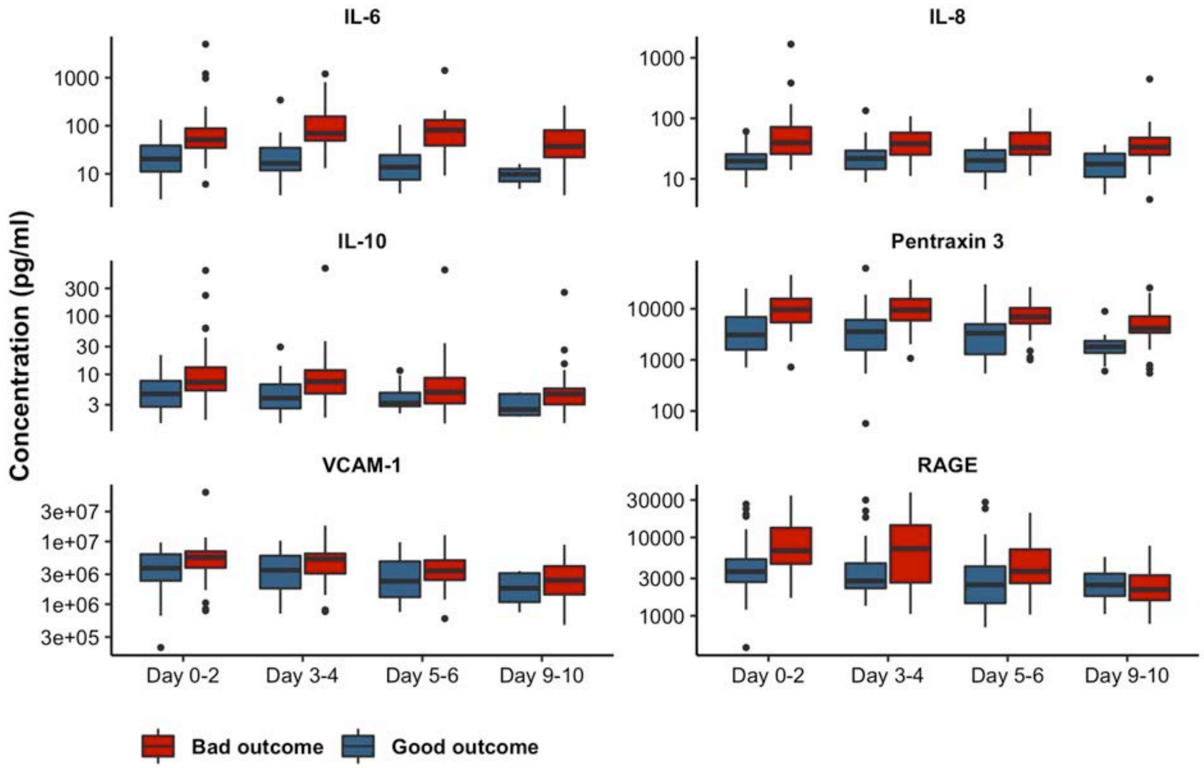

Supplemental figure 7: Fold change of biomarker concentrations during stay on the ward, stratified for favourable and non-favourable outcomes.

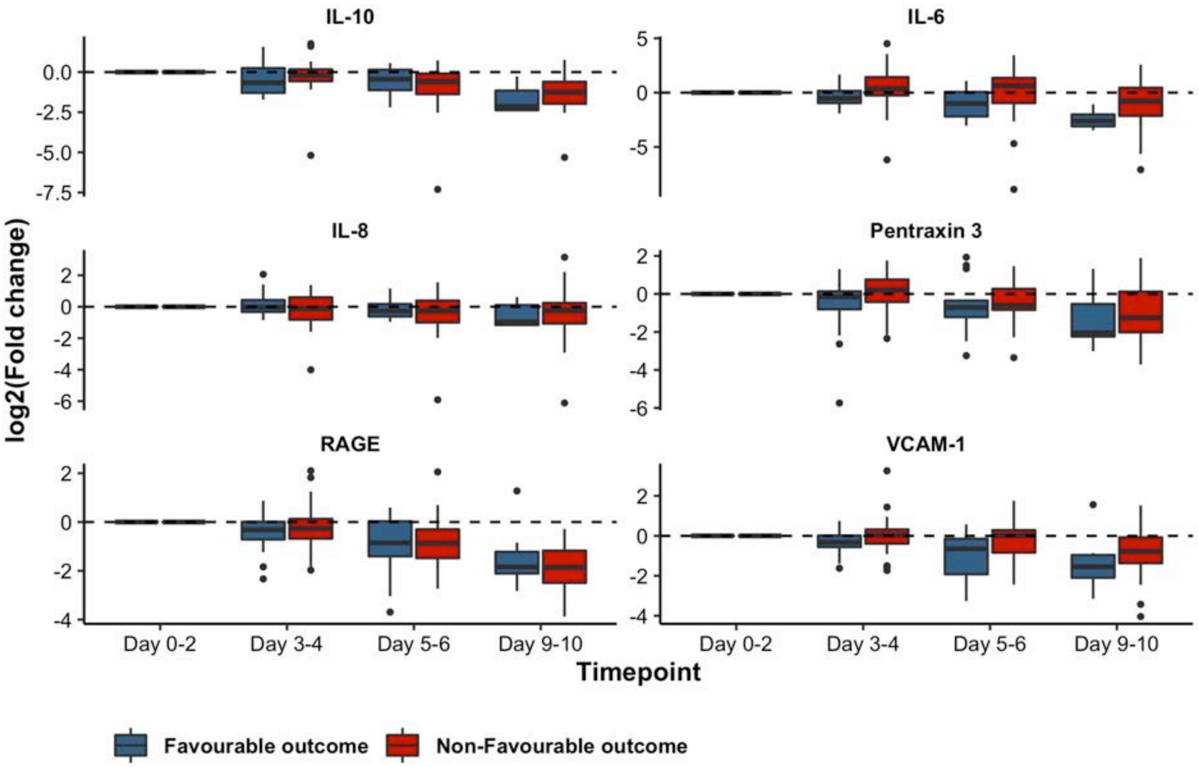

Supplemental figure 8: Biomarker concentrations during stay on the ICU, stratified for survivors vs. non-survivors

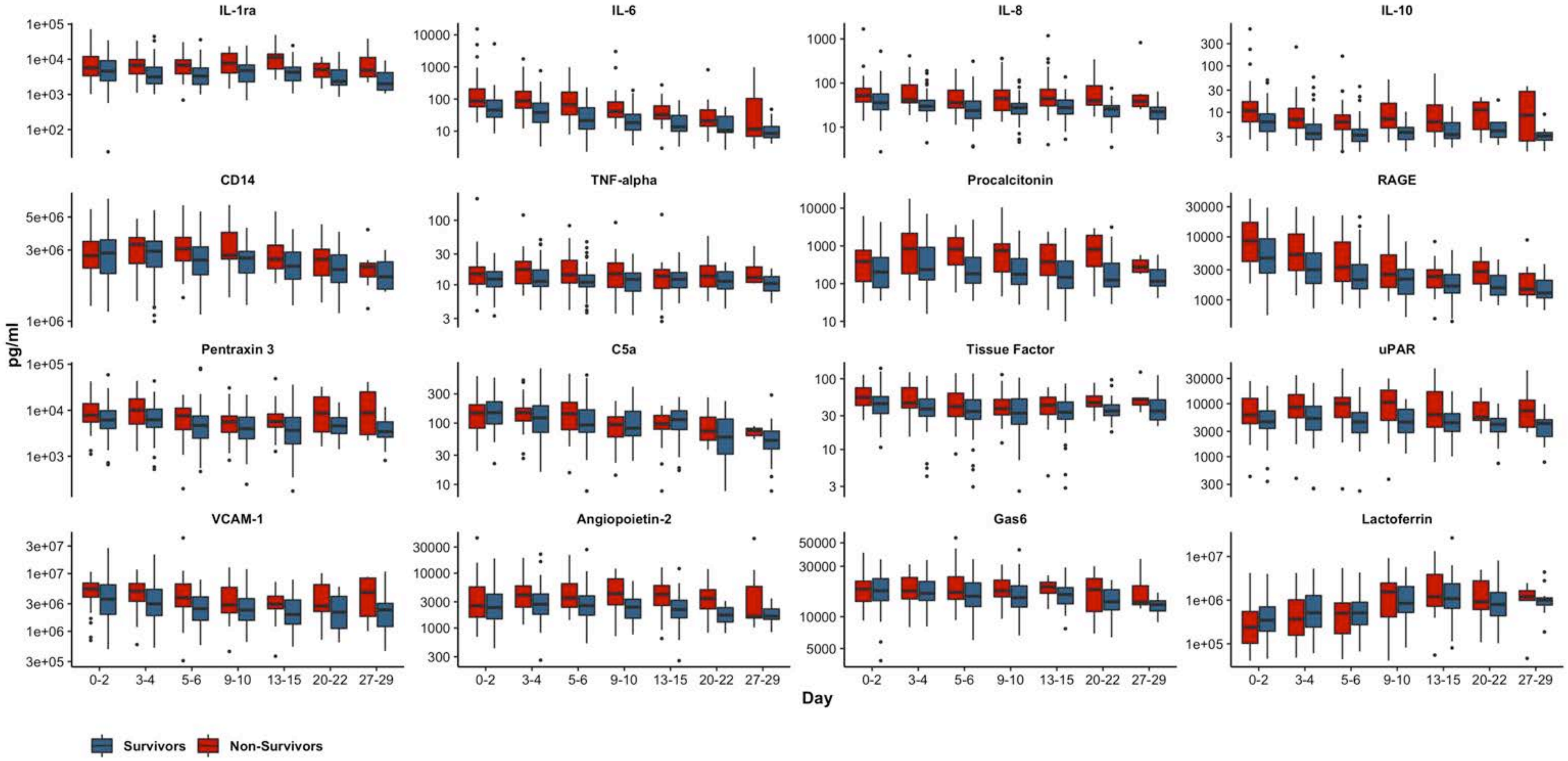

Supplemental figure 9: Fold change of biomarker concentrations during stay on the ICU, stratified for survivors vs. non-survivors

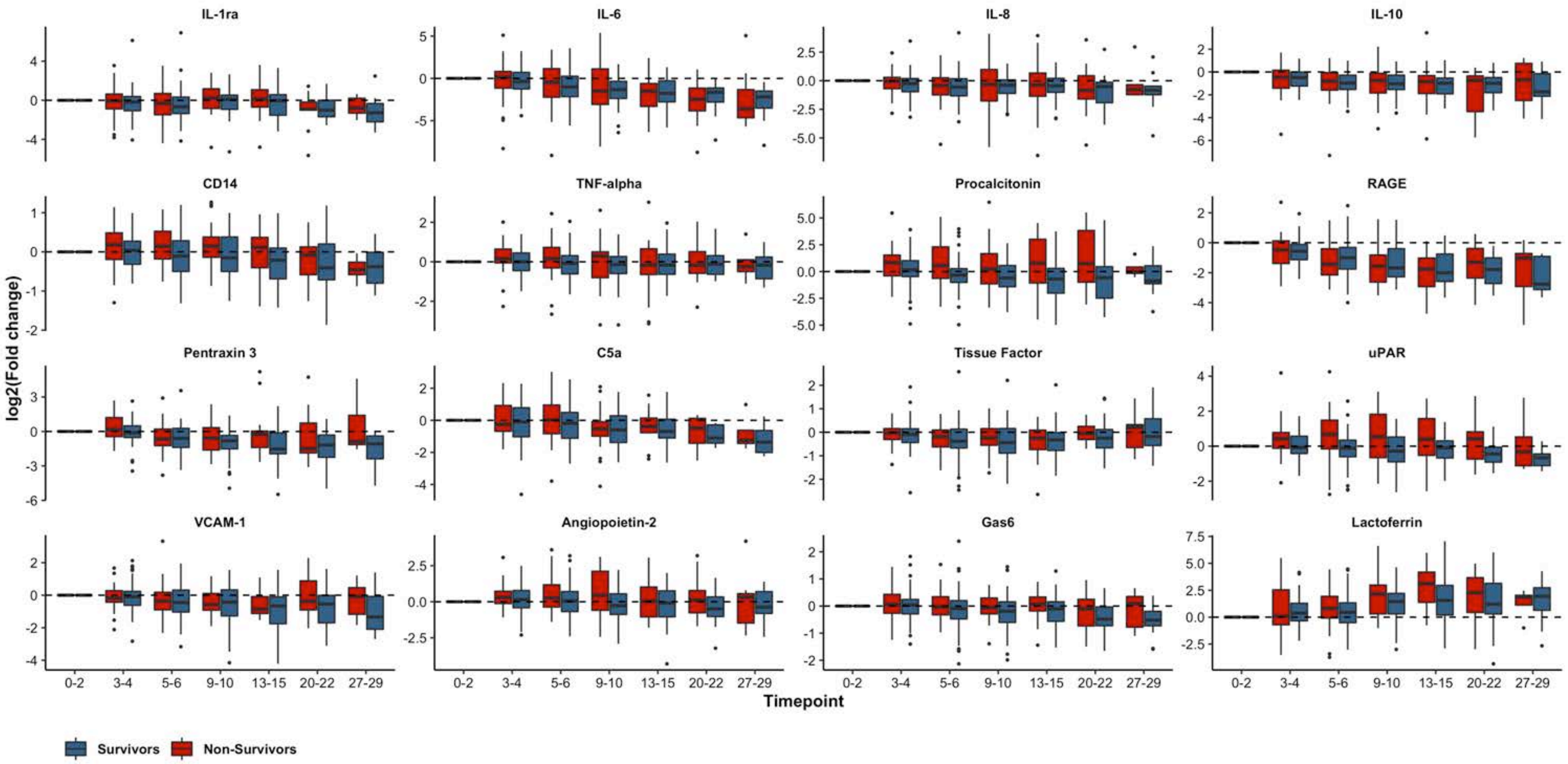

Supplemental figure 10: Odds ratio for mortality per 2-log difference in baseline concentration corrected for sex and age.

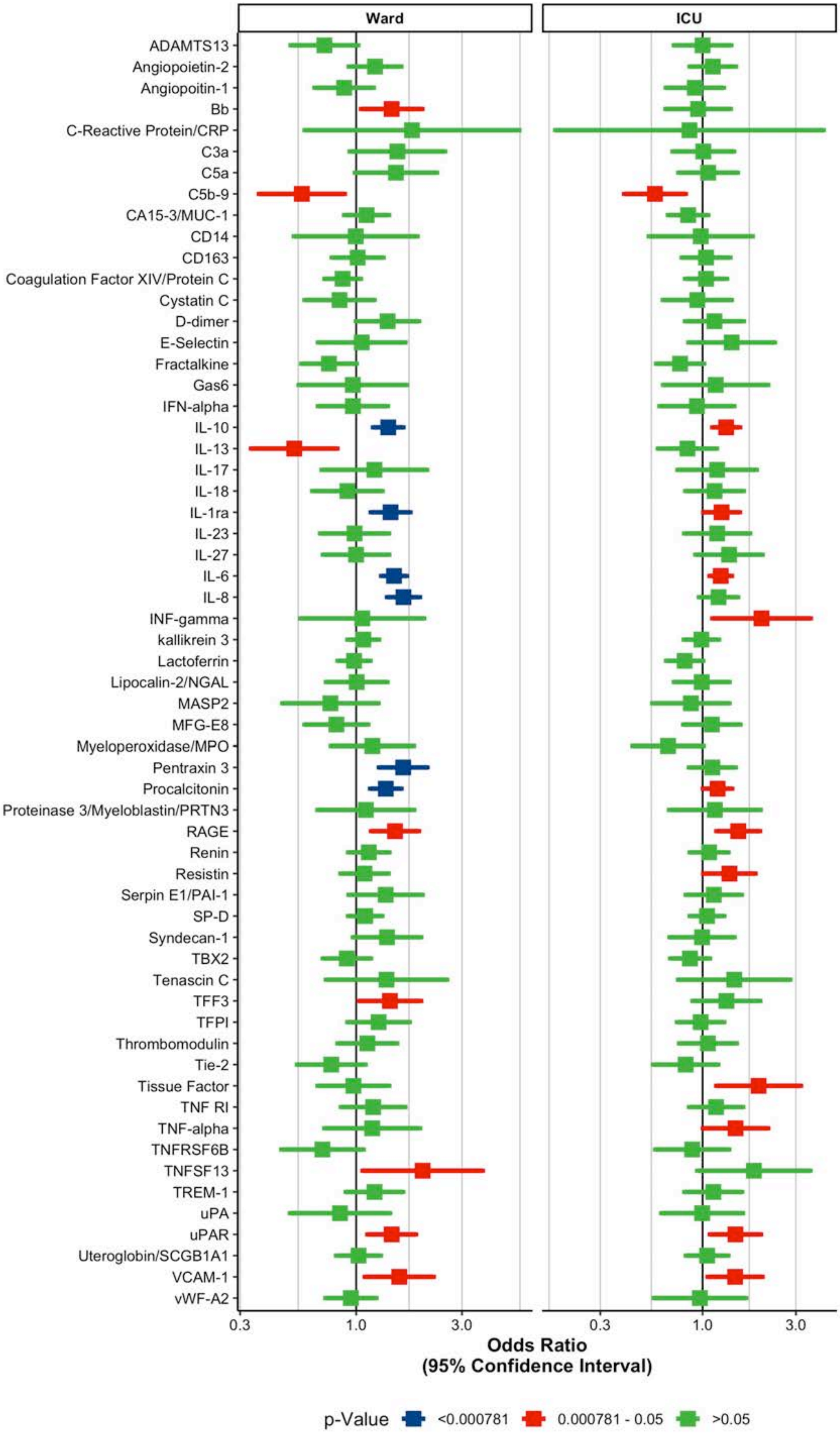

Supplemental figure 11: Odds ratio for mortality per 2-log difference in baseline concentration corrected for sex, age and without and with BMI for patients on the general ward.

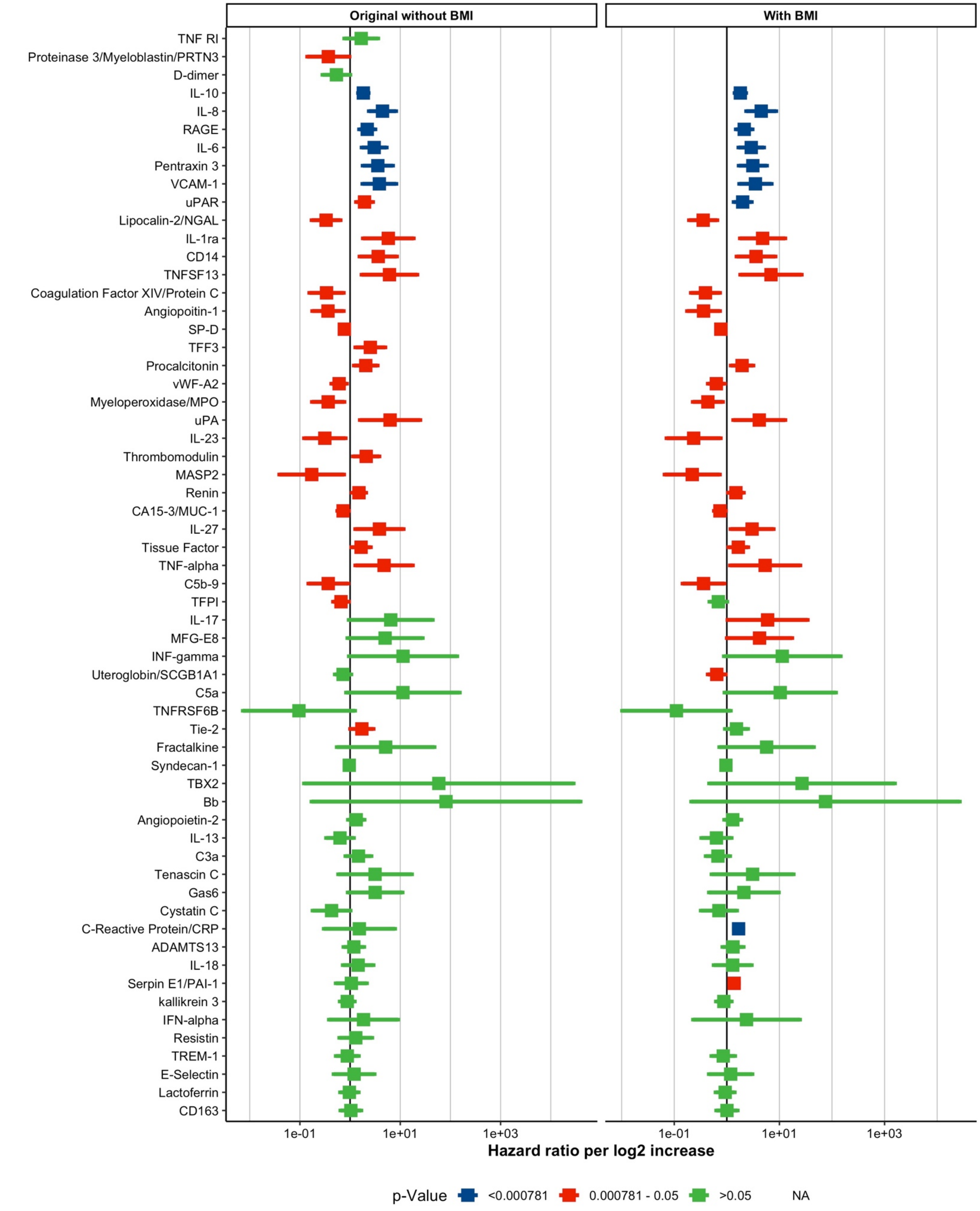

Supplemental figure 12: Odds ratio for mortality per 2-log difference in baseline concentration corrected for sex, age and without and with BMI for patients on the ICU.

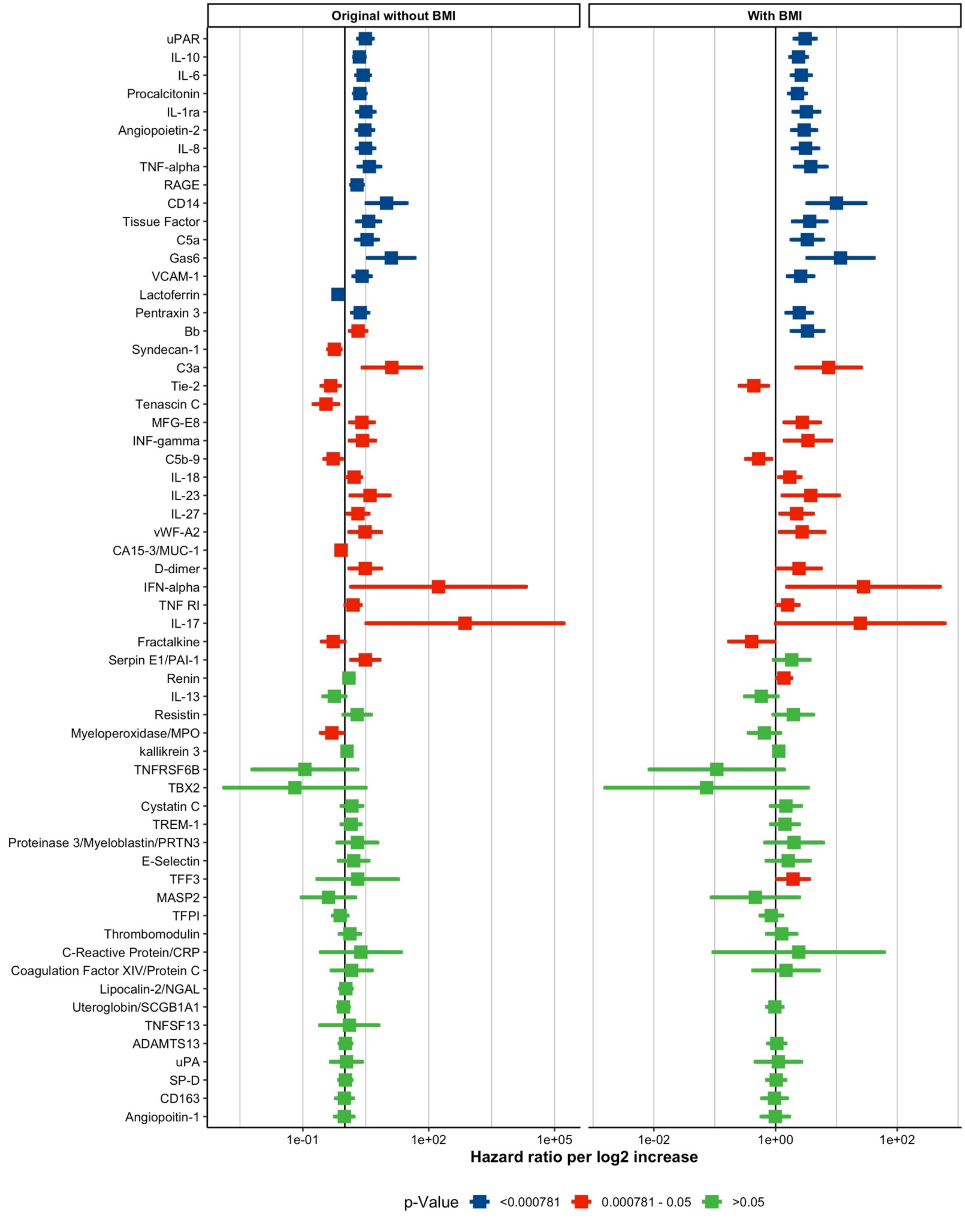

Supplemental figure 13: Association between baseline concentration of biomarkers with the occurrence of pulmonary embolism or death in the absence of pulmonary embolism, stratified per cohort. Blue is the OR for pulmonary embolism and red is for mortality.

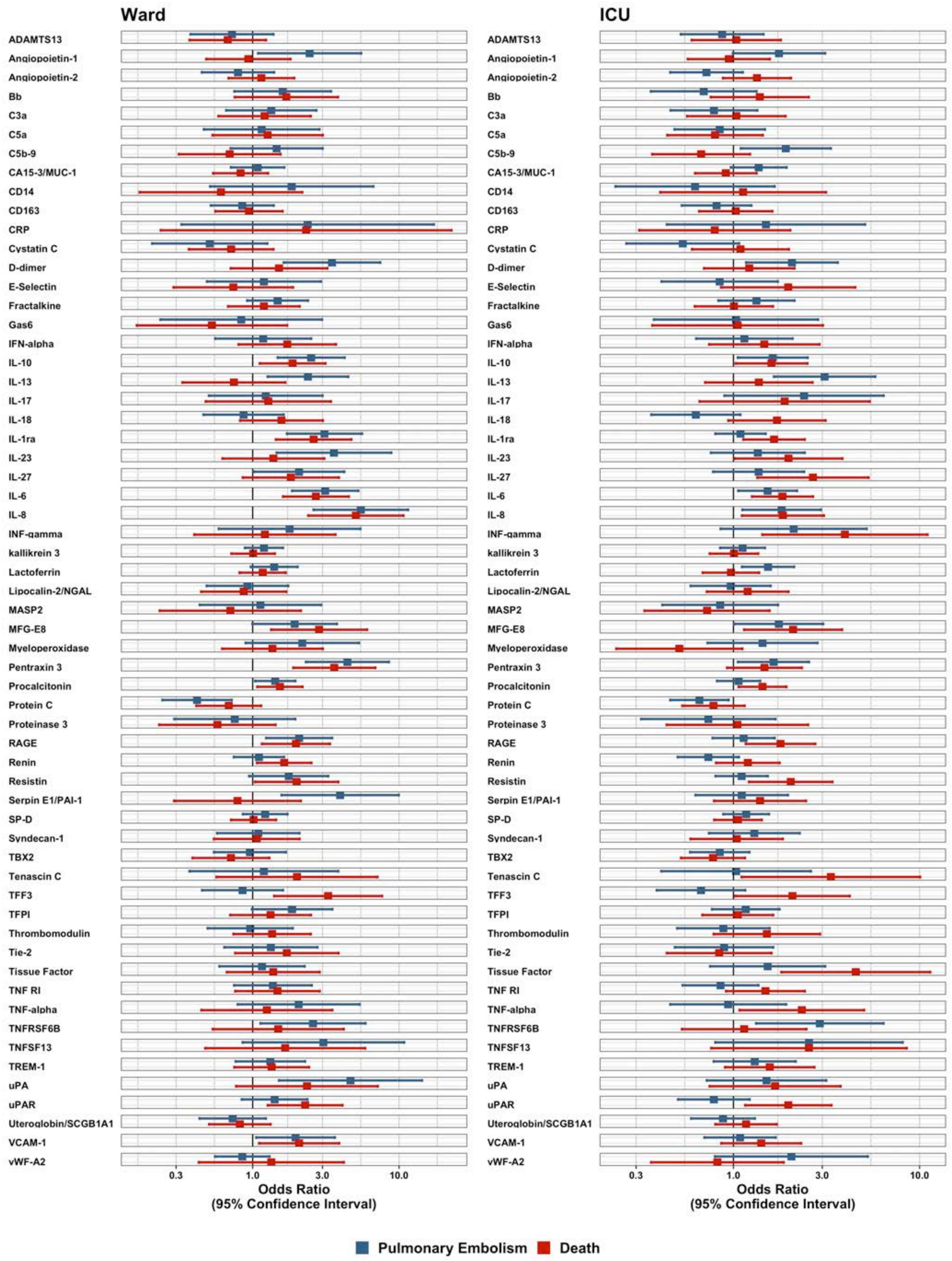

Supplemental figure 14: Association between baseline concentration of biomarkers with the occurrence of VTE or death in the absence of VTE, stratified per cohort. Blue is the OR for VTE and red is for mortality.

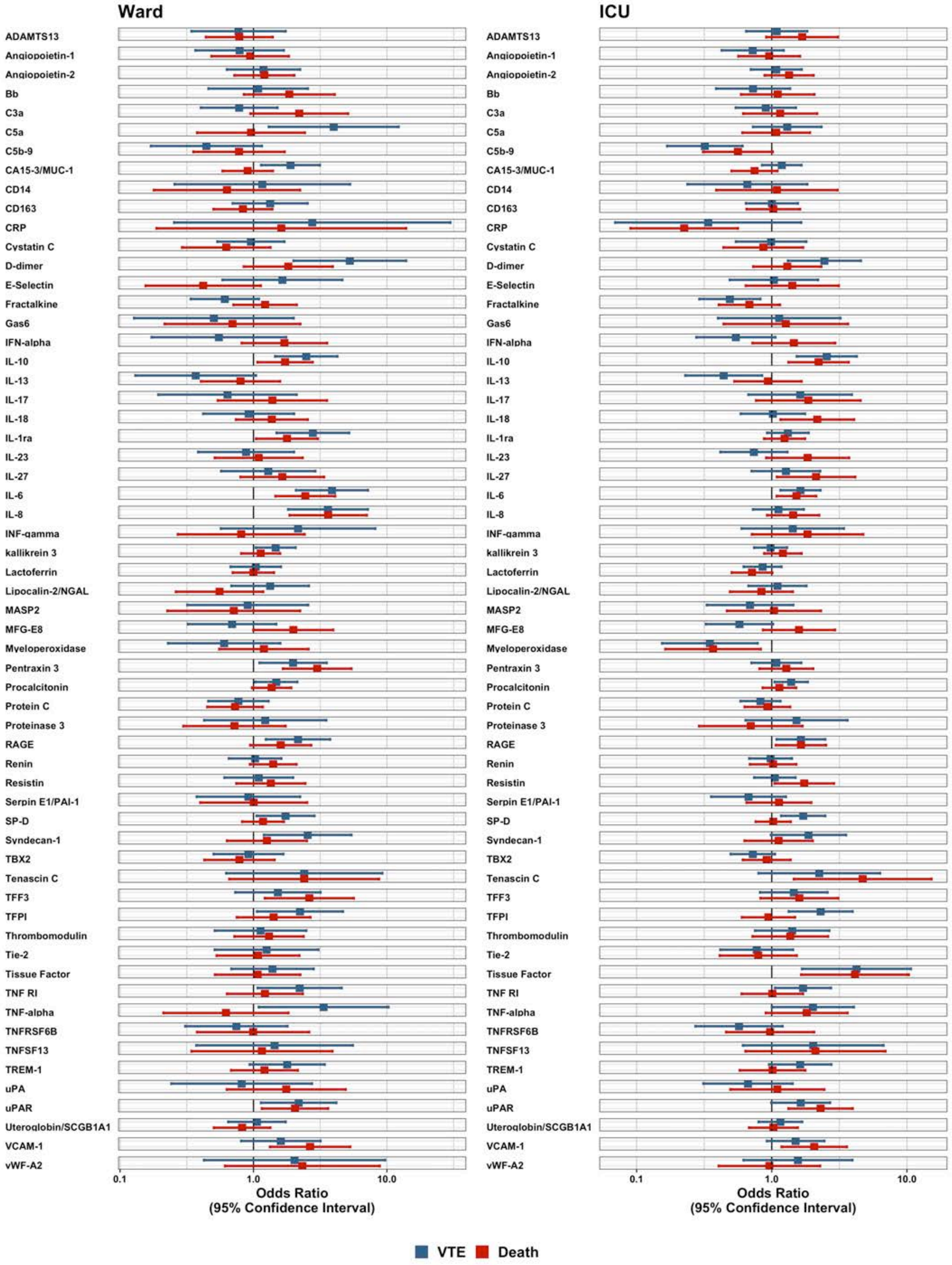

Supplemental figure 15: Association between baseline concentration of biomarkers with the occurrence of RRT or death in the absence of RRT, only shown for ICU patients. Blue is the OR for RRT and red is for mortality.

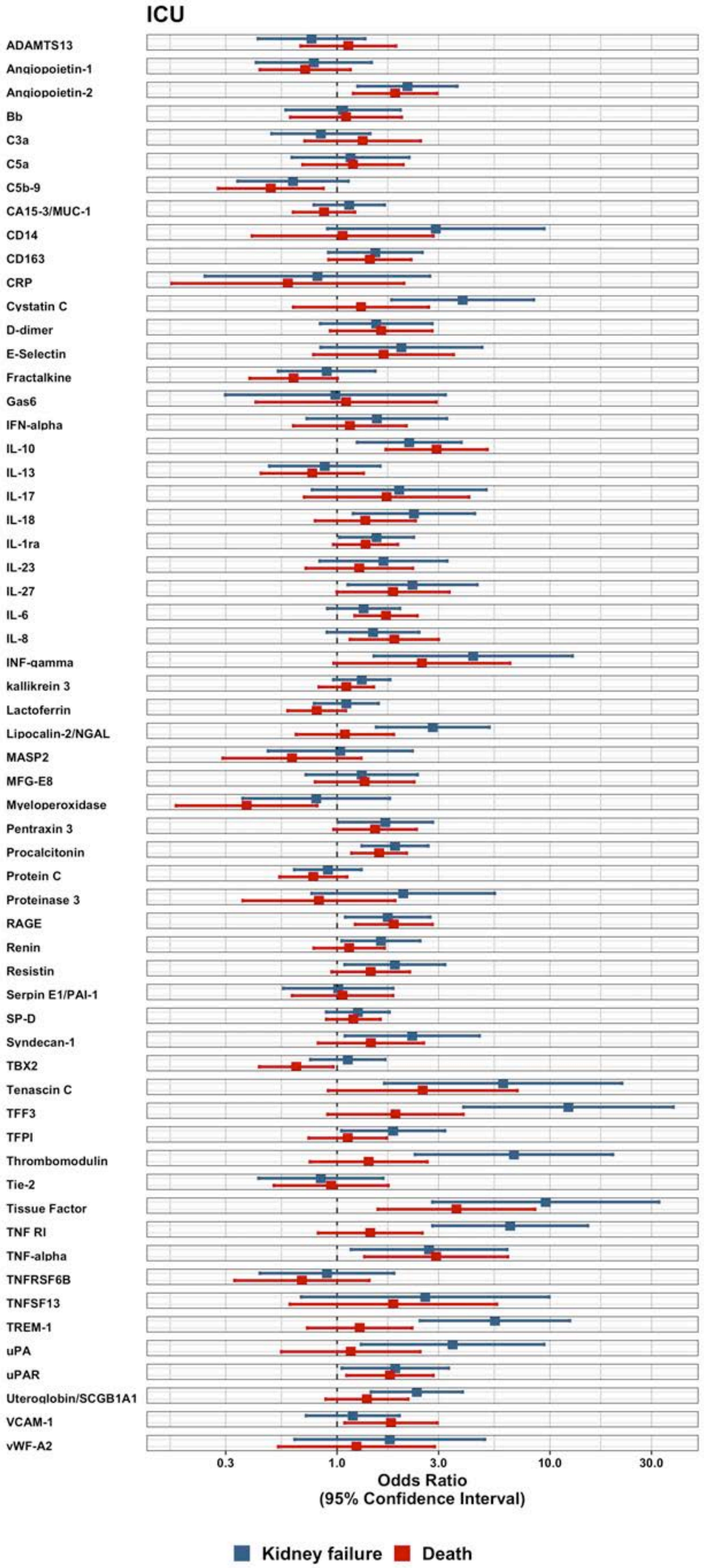

Supplemental figure 16: 60 figures with differences in biomarker concentrations between groups.

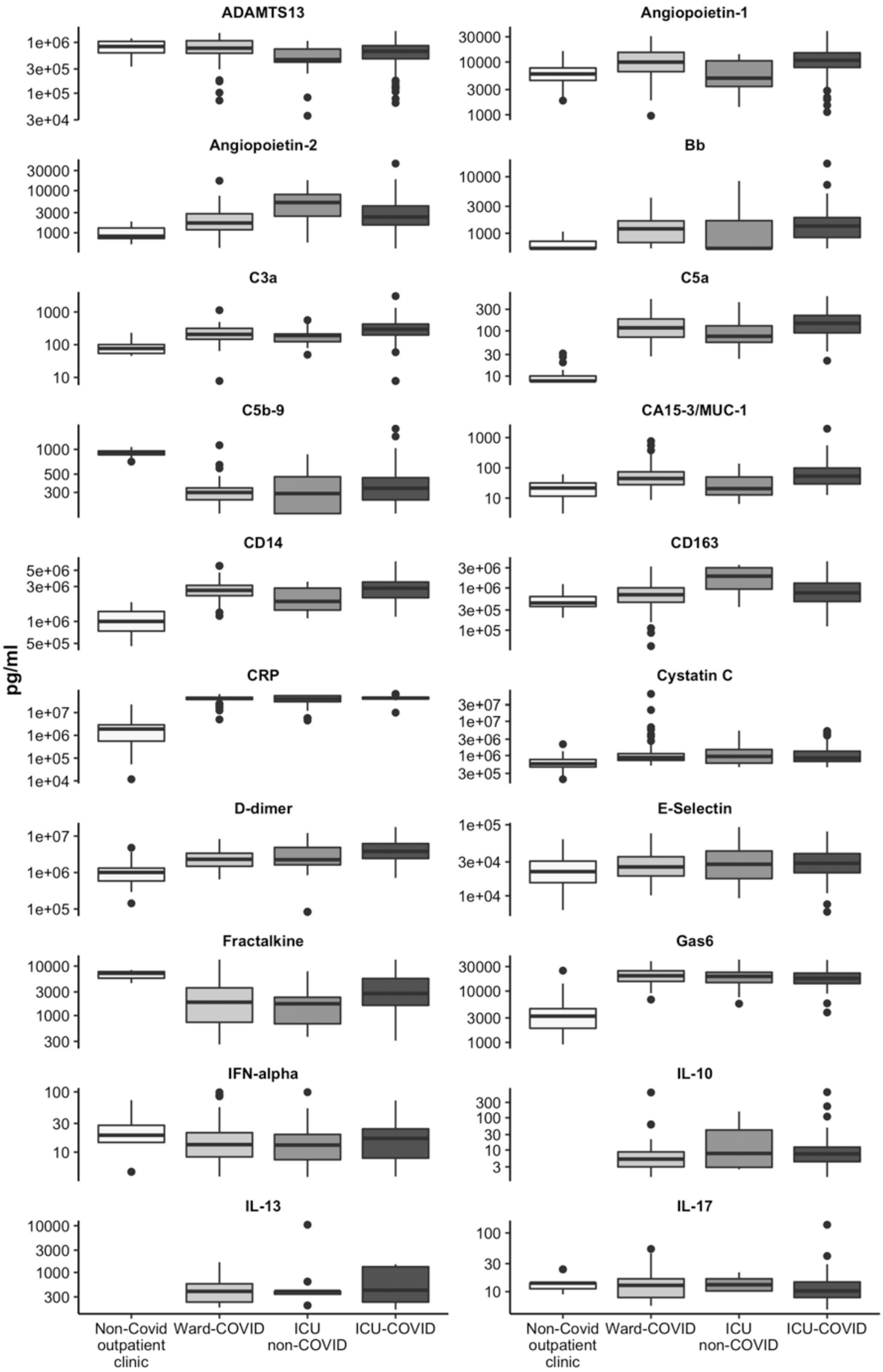

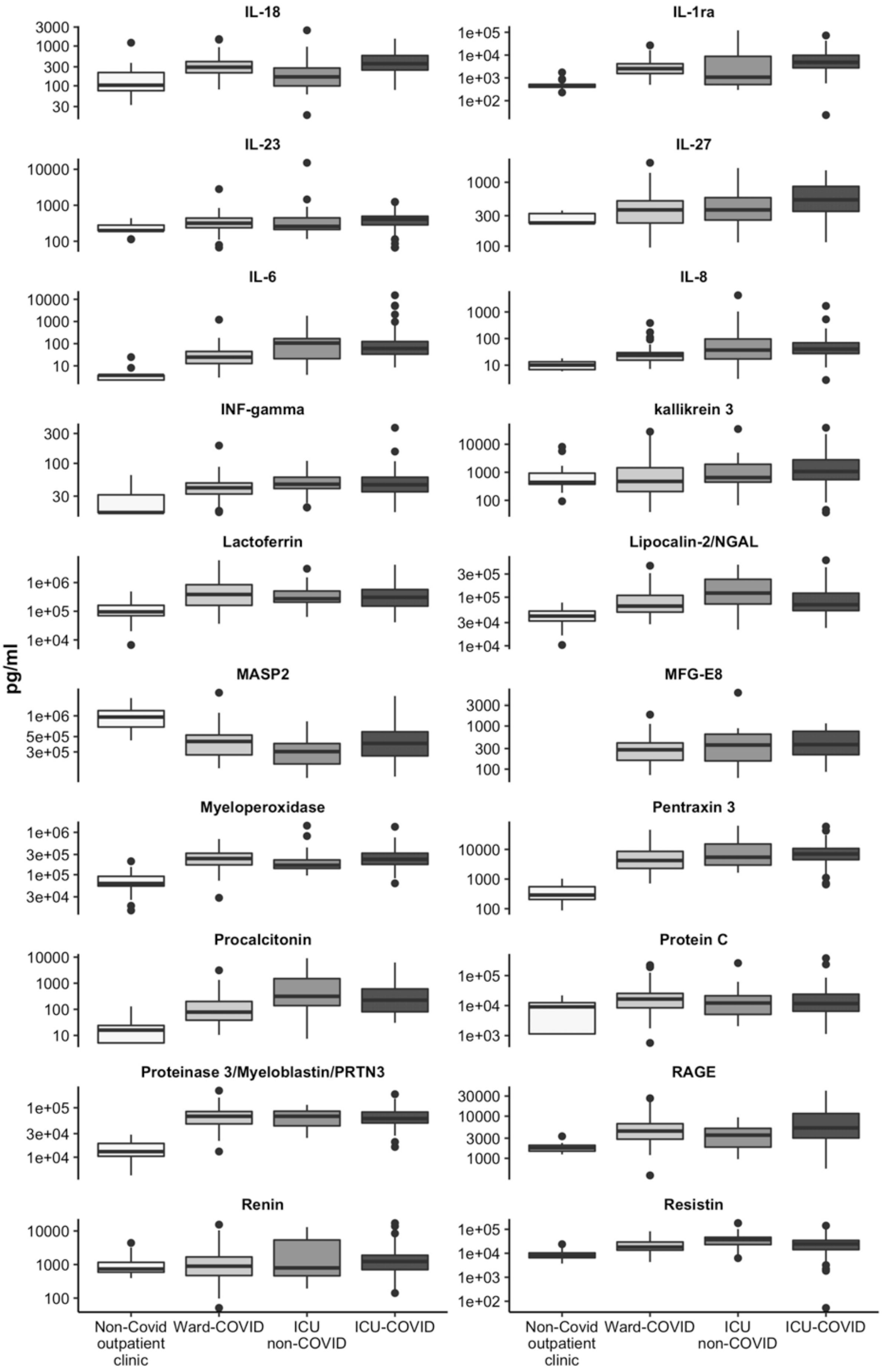
pg/ml
IL-18
IL-1ra
IL-23
IL-27
IL-6
IL-8
INF-gamma
kallikrein 3
Lactoferrin
Lipocalin-2/NGAL
MASP2
MFG-E8
Myeloperoxidase
Pentraxin 3
Procalcitonin
Protein C
Proteinase 3/Myeloblastin/PRTN3
RAGE
Renin
Resistin
Non-Covid outpatient clinic
Ward-COVID
ICU non-COVID
ICU-COVID

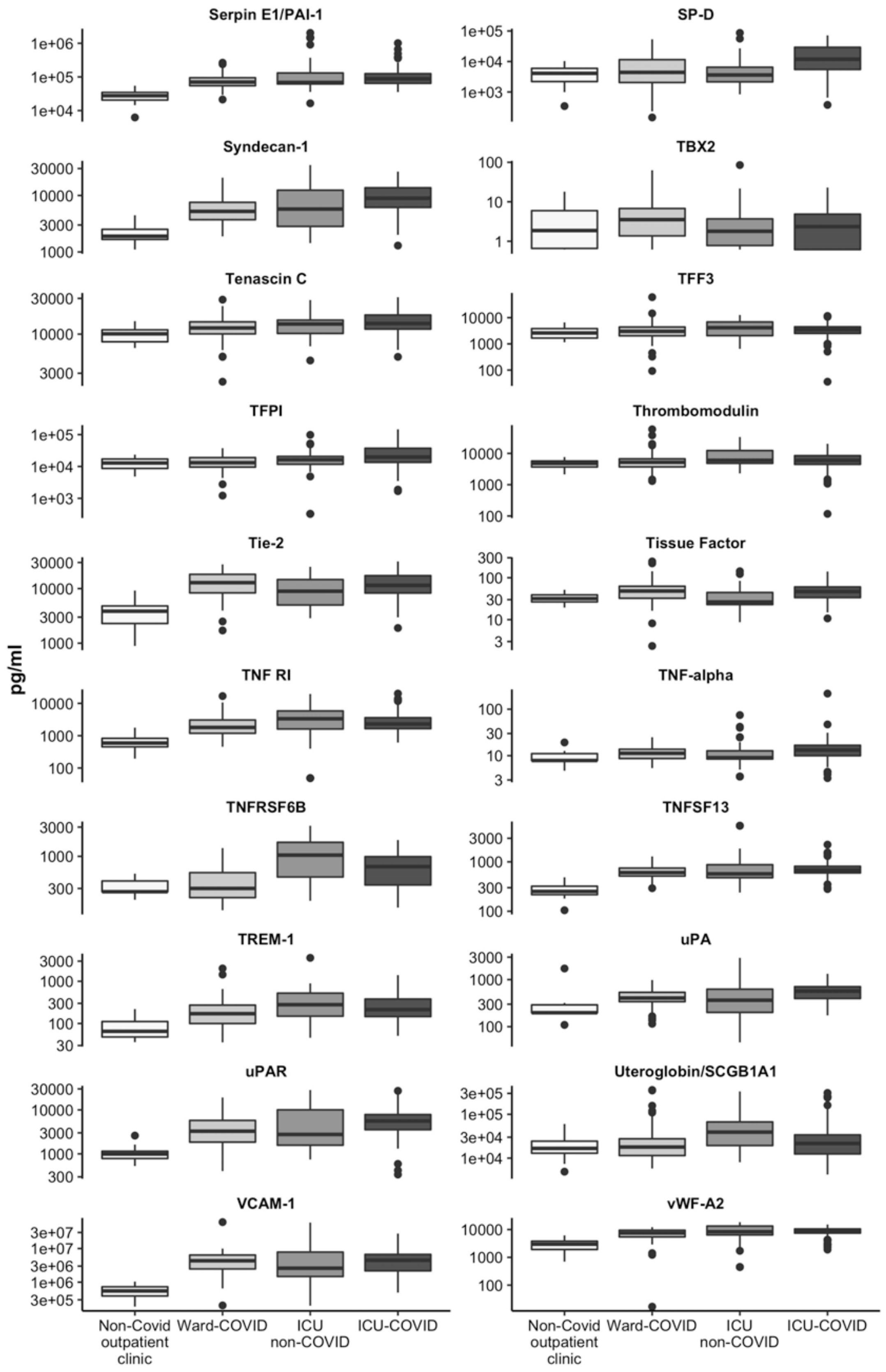

Age matched controls	Non-COVID outpatient clinic
Patients with COVID-19 admitted to the ward	Ward-COVID
Patients admitted to the ICU without COVID-19	ICU non-COVID
Patients with COVID-19 admitted to the ICU	ICU-COVID
