# Supplementary material for: Clinical features and prognostic factors in Covid-19: A prospective cohort study
Source: eBioMedicine. 2021 May 14;67:103378. doi: 10.1016/j.ebiom.2021.103378 (PMC8118723; doi:10.1016/j.ebiom.2021.103378)
Supplement: Supplementary file 4 [file mmc4.docx]

|  |  |
| --- | --- |

| **First names (will be abbreviated on Pubmed)** | **Surnames (not abbreviated)** |
| --- | --- |
| Michiel | van Agtmael |
| Anne Geke | Algera |
| Brent | Appelman |
| Frank | van Baarle |
| Diane | Bax |
| Martijn | Beudel |
| Harm Jan | Bogaard |
| Marije | Bomers |
| Peter | Bonta |
| Lieuwe | Bos |
| Michela | Botta |
| Justin | de Brabander |
| Godelieve | de Bree |
| Sanne | de Bruin |
| David T.P. | Buis |
| Marianna | Bugiani |
| Esther | Bulle |
| Osoul | Chouchane |
| Alex | Cloherty |
| David T.P. | Buis |
| Maurits C.F.J. | de Rotte |
| Mirjam | Dijkstra |
| Dave A. | Dongelmans |
| Romein W.G. | Dujardin |
| Paul | Elbers |
| Lucas | Fleuren |
| Suzanne | Geerlings |
| Theo | Geijtenbeek |
| Armand | Girbes |
| Bram | Goorhuis |
| Martin P. | Grobusch |
| Florianne | Hafkamp |
| Laura | Hagens |
| Jorg | Hamann |
| Vanessa | Harris |
| Robert | Hemke |
| Sabine M. | Hermans |
| Leo | Heunks |
| Markus | Hollmann |
| Janneke | Horn |
| Joppe W. | Hovius |
| Menno D. | de Jong |
| Rutger | Koning |
| Endry H.T. | Lim |
| Niels | van Mourik |
| Jeannine | Nellen |
| Esther J. | Nossent |
| Frederique | Paulus |
| Edgar | Peters |
| Dan A.I. | Piña-Fuentes |
| Tom | van der Poll |
| Bennedikt | Preckel |
| Jan M. | Prins |
| Jorinde | Raasveld |
| Tom | Reijnders |
| Michiel | Schinkel |
| Femke A.P. | Schrauwen |
| Marcus J. | Schultz |
| Alex | Schuurmans |
| Jaap | Schuurmans |
| Kim | Sigaloff |
| Marleen A. | Slim |
| Patrick | Smeele |
| Marry | Smit |
| Cornelis S. | Stijnis |
| Willemke | Stilma |
| Charlotte | Teunissen |
| Patrick | Thoral |
| Anissa M. | Tsonas |
| Pieter R. | Tuinman |
| Marc | van der Valk |
| Denise | Veelo |
| Carolien | Volleman |
| Heder | de Vries |
| Lonneke A. | Vught |
| Michèle | van Vugt |
| Dorien | Wouters |
| A. H (Koos) | Zwinderman |
| Matthijs C. | Brouwer |
| W. Joost | Wiersinga |
| Alexander P.J. | Vlaar |
| Diederik | van de Beek |
