# Supplementary material for: Clinical features and prognostic factors in Covid-19: A prospective cohort study
Source: eBioMedicine. 2021 May 14;67:103378. doi: 10.1016/j.ebiom.2021.103378 (PMC8118723; doi:10.1016/j.ebiom.2021.103378)
Supplement: Supplementary file 5 [file mmc5.docx]

Michiel van Agtmael^2^, Anne Geke Algera^1^, Brent Appelman^2^, Frank van Baarle^1^, Diane Bax^3^, Martijn Beudel^4^, Harm Jan Bogaard^5^, Marije Bomers^2^ Peter Bonta^5^, Lieuwe Bos^1^, Michela Botta^1^, Justin de Brabander^2^, Godelieve de Bree^2^, Sanne de Bruin^1^, David T.P. Buis^1^, Marianna Bugiani^5^, Esther Bulle^1^, Nora Chekrouni^4^, Osoul Chouchane^2^ Alex Cloherty^3^, Mirjam Dijkstra^12^, Dave A. Dongelmans^1^, Romein W.G. Dujardin^1^, Paul Elbers^1^, Lucas Fleuren^1^, Suzanne Geerlings^2^ Theo Geijtenbeek^3^, Armand Girbes^1^, Bram Goorhuis^2^, Martin P. Grobusch^2^, Florianne Hafkamp^3^, Laura Hagens^1^, Jorg Hamann^7^, Vanessa Harris^2^, Robert Hemke^8^, Sabine M. Hermans^2^ Leo Heunks^1^, Markus Hollmann^6^, Janneke Horn^1^, Joppe W. Hovius^2^, Menno D. de Jong^9^, Rutger Koning^4^, Endry H.T. Lim^1^, Niels van Mourik^1^, Jeaninne Nellen^2^, Esther J. Nossent^5^, Sabine Olie^4^, Frederique Paulus^1^, Edgar Peters^2^, Dan A.I. Pina-Fuentes^4^, Tom van der Poll^2^, Bennedikt Preckel^6^, Jan M. Prins^2^, Jorinde Raasveld^1^, Tom Reijnders^2^, Maurits C.F.J. de Rotte^12^, Michiel Schinkel^2^, Marcus J. Schultz^1^, Femke A.P. Schrauwen^12^, Alex Schuurmans^10^, Jaap Schuurmans^1^, Kim Sigaloff^1^, Marleen A. Slim^1,2^, Patrick Smeele^5^, Marry Smit^1^, Cornelis S. Stijnis^2^, Willemke Stilma^1^, Charlotte Teunissen^11^, Patrick Thoral^1^, Anissa M Tsonas^1^, Pieter R. Tuinman^2^, Marc van der Valk^2^, Denise Veelo^6^, Carolien Volleman^1^, Heder de Vries^1^, Lonneke A. Vught^1,2^, Michèle van Vugt^2^, Dorien Wouters^12^, A. H (Koos) Zwinderman^13^, Matthijs C. Brouwer^4^, W. Joost Wiersinga^2^, Alexander P.J. Vlaar^1^, Diederik van de Beek^4^.

^1^Department of Intensive Care, Amsterdam UMC, Amsterdam, The Netherlands; ^2^Department of Infectious Diseases, Amsterdam UMC, Amsterdam, The Netherlands; ^3^Experimental Immunology, Amsterdam UMC, Amsterdam, The Netherlands; ^4^Department of Neurology, Amsterdam UMC, Amsterdam, The Netherlands; ^5^Department of Pulmonology, Amsterdam UMC, Amsterdam, The Netherlands; ^6^Department of Anesthesiology, Amsterdam UMC, Amsterdam, The Netherlands; ^7^Amsterdam UMC Biobank Core Facility, Amsterdam UMC, Amsterdam, The Netherlands; ^8^Department of Radiology, Amsterdam UMC, Amsterdam, The Netherlands; ^9^Department of Medical Microbiology, Amsterdam UMC, Amsterdam, The Netherlands; ^10^Department of Internal Medicine, Amsterdam UMC, Amsterdam, The Netherlands; ^11^Neurochemical Laboratory, Amsterdam UMC, Amsterdam, The Netherlands; ^12^Department of Clinical Chemistry, Amsterdam UMC, Amsterdam, The Netherlands; ^13^Department of Clinical Epidemiology, Biostatistics and Bioinformatics, Amsterdam UMC, Amsterdam, The Netherlands.
